# Molecular Dynamics Simulations of Electric Field Poled Poly(methyl methacrylate) Doped with Tricyanopyrroline Chromophores

**DOI:** 10.1021/acs.jpcb.5c02832

**Published:** 2025-07-25

**Authors:** Nils M. Denda, Erik Rohloff, Florens R. Kurth, Li Zhao, Hans-Hermann Johannes, Wolfgang Kowalsky, Carolin König, Peter Behrens, Andreas M. Schneider

**Affiliations:** † Institute of Inorganic Chemistry, 26555 Leibniz University Hannover, Hannover 30167, Germany; ‡ Cluster of Excellence PhoenixD (Photonics, Optics, and Engineering - Innovation Across Disciplines), Leibniz University Hannover, Hannover 30167, Germany; § Institute of High-Frequency Technology, 26527 Technische Universität Braunschweig, Braunschweig 38106, Germany; ∥ Institute of Physical Chemistry and Electrochemistry, Leibniz University Hannover, Hannover 30167, Germany

## Abstract

Nonlinear optical polymer-based materials are promising candidates for future high-bandwidth data processing and other optically driven applications. This class of materials consists of noncentrosymmetrically aligned electro-optic (EO) active chromophores embedded in a polymer matrix. However, there are no experimental measurement methods available to directly investigate the molecular orientation of individual chromophore molecules in the polymer matrix. Therefore, a reliable simulation protocol was developed to fill this gap. This study extends previous work in the context of larger atomistic polymer models and a contemporary chromophore molecule. In contrast to earlier approaches, a quantum mechanical continuum solvation method is introduced to account for local field effects. The experimentally accessible EO tensor element *r*_33_ is calculated and contextualized with other studies; good agreement with experimental values is demonstrated. We provide a comprehensive molecular simulation protocol for the design, development, and analysis of novel materials.

## Graphical abstract

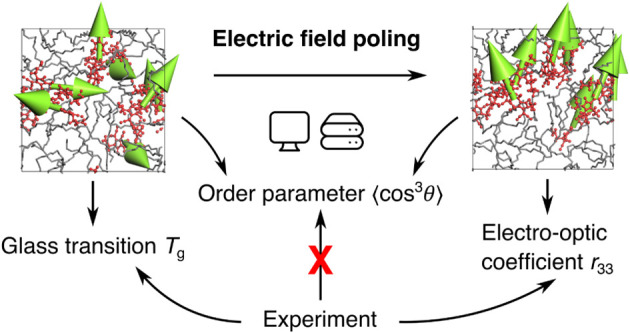

## Introduction

Host–guest materials, consisting of a polymer as a host and electro-optic (EO) active chromophore molecules as guests, are economically promising and easily processable materials for future telecommunication and optics applications compared to conventional EO materials like crystalline lithium niobate LiNbO_3_. The chromophore molecules often consist of an electron donor–acceptor structure with highly polarizable π electrons. If the dipoles of the chromophores are aligned in parallel and the resulting noncentrosymmetrical order is stabilized in the vitrified polymer matrix, macroscopic EO effects like the linear Pockels effect can be exploited. A low-frequency electric field (0 THz to 10 THz, with respect to the optical field) allows to modulate the macroscopic electromagnetic susceptibility of the material and, therefore, the refractive index (RI).^1^

These host–guest materials are seminal for applications like tunable Fabry-Pérot interferometers (etalons) for optical ultrasound detection, tunable optical filters, high-resolution spectrometers, and spatial light modulators in the visible range.^2,3^ Photonic platforms for neural probes in optogenetics and other on-chip devices for telecommunications, computing, detection, terahertz generation, and construction of functional photonic crystals are further potential applications for EO polymer materials.^4,5^ EO modulators on optical platforms enable data processing and conversion of electronic signals into optical signals (e.g., as in an electrocardiogram).^4,6^ Integrating these modulators as silicon-organic hybrid materials in silicon photonics is another promising prospect.^7–9^ Polymer-based EO materials have many advantages regarding bandwidth, dielectric constant, half-wave voltage, EO coefficients, processability, microstructuring, and, last but not least, material cost.^5^ Future applications may include large-scale gigahertz switching networks for quantum photonics and photonic neural networks.^10^

The molecular design of chromophores and the corresponding polymer system is crucial for poling efficiency, maximizing the EO effect, and the long-term stability of the chromophore alignment.^5^ The optimal host–guest design is still challenging and subject of current research.^5^ The experimental investigations on host–guest materials have a long history. However, there are only a few conceptual molecular modeling studies on rather simple chromophores and small model systems of these complex hybrid materials (Monte Carlo-based investigations^11–14^ and molecular dynamics-based investigations^15–21^ ). This is surprising since theoretical investigations of molecular interdependencies and the insights on the molecular scale can improve the development of new materials and promote the accelerated screening of promising host and guest candidates.

The first Monte Carlo (MC) statistical mechanical simulation approaches were elaborated by Dalton and Robinson^11–13^ and continued by Robinson and Tillack.^14^ At that time, coarse-grained chromophore models and MC lattice simulations were computationally feasible. The alignment process and influence of the chromophore shape and chromophore concentration were the subjects of these studies. Even very large dendrimer chromophores were investigated with certain substantial simplifications.^13^

Fully atomistic molecular dynamics (MD) simulations of both the chromophore and the polymer matrix were first performed by Kim and Hayden^15^ in 1999. At that time, only small chromophore molecules embedded in small-sized host systems could be treated, e.g., two chromophore molecules (*N,N*-dimethyl-*p*-nitroaniline) distributed in 90 repeat units of poly­(methyl methacrylate). A few years later, Makowska-Janusik et al.^16^ investigated larger chromophore molecules with distinct molecular flexibility and different shapes.^16^

Leahy-Hoppa et al.^17^ were the first authors to assess the concentration dependence of chromophores for the poling process at an atomistic level.

In 2007, Ågren and coworkers were the first to put simulation results of the molecular scale in the context of the macroscopically, experimentally determined EO tensor element *r*_33_, providing a direct link to experimental investigations and results.^18,19^ With increased computing resources, lower field strengths, longer equilibration or simulation times, as well as extended host–guest models with different chromophore concentrations were computational feasible. Tu et al.^18^ used a field strength 2 orders of magnitude smaller than Kim and Hayden.^15^ Moreover, MD simulations with simulation times of up to 30 ns were feasible compared to only a few nanoseconds in ref 15. Last but not least, in 2007, more chromophores were incorporated (32 to 96 instead of only two), allowing for greater flexibility in the design of structure models.

Following this trend, we aim to explore the capabilities of modern hardware and molecular modeling methods in even larger systems in order to advance today’s material research. In this study, we investigate the chromophore “C3”, a tricyanopyrroline derivative with a strong acceptor group (4-cyano-5-dicyanomethylene-2-oxo-3-pyrrolin-3-yl group, abbreviated as the TCP group) (Figure 1). Although the TCP group has been known since the 1960s, it is still of interest for novel host–guest materials because of its outstanding electron acceptor character.^22^

**Figure 1:**
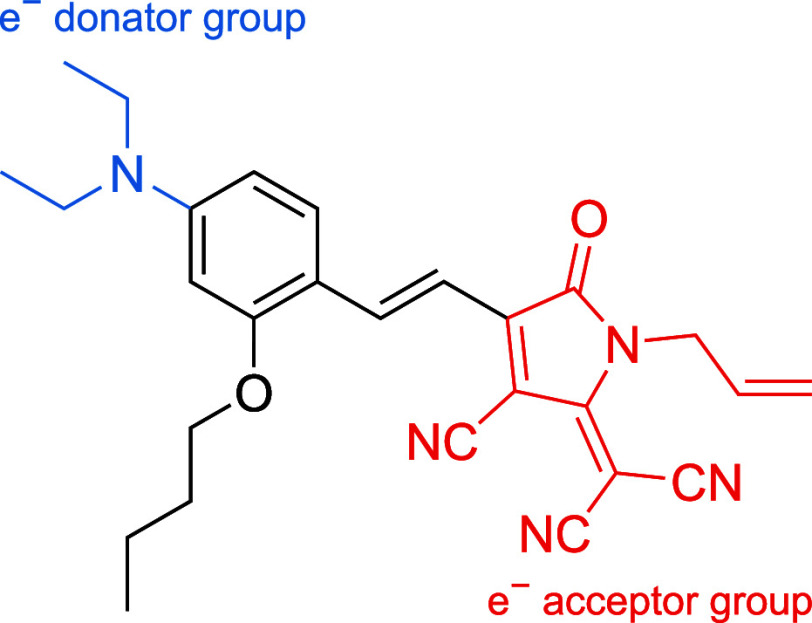
Chromophore “C3”, a tricyanopyrroline derivative studied in this work (TCP group, red highlighted).

Based on previously developed molecular modeling methods,^18,19^ the novel “C3” chromophore is investigated with polarizable continuum model methods on the quantum chemistry level. Host–guest models are developed on an atomistic level and investigated with force field methods. We provide a rational methodology exploiting model sizes and time scales as much as necessary for reliable results. In the first approach, we adopt the local field factor-based *r*_33_ estimation procedure developed by Tu et al.^18^ and Zhang et al.^19^ In the second step, we include a quantum mechanical continuum solvation method replacing local field factors, resulting in a more straightforward *r*_33_ calculation. Electro-optic coefficients and optical properties of both approaches are contextualized with experimental values and other simulation studies, showing the reliability of the presented protocol.

## Computational Methods and Experimental Details

All force field (FF) calculations were performed in the BIOVIA Materials Studio 23.1 ^23^ environment using the COMPASS III force field.^24^ Polymer start structures and host–guest models were generated using the Amorphous Cell module. Energy minimization (EM), molecular dynamics (MD) simulations, and electric field poling were conducted in the Forcite module. Chromophore geometry optimization and coupled-perturbed Hartree–Fock (CPHF) calculations were performed in Gaussian 16, Revision B.01.^25^

### Chromophore Characterization

The C3 chromophore structure was energy minimized by employing FF methods. Subsequently, the molecular structure was further optimized using a density functional theory (DFT) approach (B3LYP/aug-cc-pVTZ).^26–30^ Atomic charges were obtained via the Merz–Kollman^31,32^ electrostatic potential (ESP) fitting method. To obtain (hyper)­polarizability values, CPHF^33–35^ calculations on DFT level with a range-separated functional (CAM-B3LYP^36^/aug-cc-pVTZ) were performed. DFT polarizability values were calculated in a solvent reaction field in Gaussian (PCM, polarizable continuum model)^37–39^ to account for effects arising from the polymer environment. Diethylamine (*ε* = 3.5766) was chosen as the solvent because its permittivity is similar to the low-frequency permittivity of poly­(methyl methacrylate) (*ε* = 3.6 ^40^). In this article, all values of the permittivity are related to the vacuum permittivity and are expressed without units. All DFT calculations described above give results comparable to post-Hartree–Fock methods, such as second-order Møller–Plesset perturbation (MP2) theory,^41,42^ enabling the application of a large-sized basis set on large chromophore molecules, e.g., chromophore C3.

Simulations using default COMPASS III FF atomic charge parameters yield a significantly lower dipole moment compared with DFT calculations. This observation is commonly known from previous molecular modeling studies.^15,17–19^ To ensure proper behavior of the chromophore in the applied electric field, atomic charges derived from DFT calculations are employed.

### Polymer Model and Host–Guest System Development

Calculated polymer-related properties, e.g., the glass transition, are highly dependent on the model size. To achieve a good trade-off between computational effort and accurate representation, two different polymer model approaches are established: The first one focuses on larger-scaled polymer models (referred to as large model) to obtain reliable and reproducible results for the glass transition, whereas the second one focuses on midsized polymer models (referred to as small model) to enable the exploration of larger time scales and to ensure proper relaxation, e.g., chromophore alignment in the host polymer. Midsized host–guest models also enable fast and parallel mass production of MD trajectories to develop statistics of chromophore alignment.

First, atactic poly­(methyl methacrylate) (PMMA) chains with a length of 100 repeat units (RU) were built with the Polymer Builder and energy-minimized. Afterward, different polymer models were constructed with the frequently used^15,18,19,21^ Amorphous Cell module. The algorithm is Monte Carlo-based and successively grows polymer chains in the unit cell. In this process, polymer backbone torsions are varied to fit torsional distribution functions typically observed in polymer structures. Finally, chromophore molecules were incorporated randomly into the chains. For the resulting models, target temperature and density are chosen to be 298 K and 1.27 gcm^–3^. The chosen target density is slightly higher than the experimental density to obtain a dense host–guest structure (compare ref 18). All model compositions are listed in Table 1.

**Table 1:** Model Compositions (RU = Repeat Units of PMMA)

Amount of C3	Small model: 3 × 100 RU + #C3	#Atoms	Large model: 14 × 100 RU + #C3	#Atoms
0 wt%	0	4506	0	21,028
10 wt%	7	4947	34	23,170
15 wt%	12	5262	–	–
20 wt%	16	5514	77	25,879
25 wt%	22	5892	–	–
30 wt%	28	6270	132	29,344

Only a few large models are constructed for glass transition analysis due to the significant amount of computation time and the large number of attempts required for successful model creation. Glass transition analyses were also performed for the small polymer models, resulting in more fluctuating values with larger uncertainties. The small models were utilized for poling and relaxation simulations on larger time scales. Nine independent models were constructed for each mass concentration. The building process for these models was much faster and more feasible than for the large models, making it suitable for massive parallelization.

To relax unfavorable close contacts, an energy minimization is performed, followed by quench dynamics (QD) simulation after the building process. A QD simulation combines an MD simulation with additional energy minimization steps after a specific number of time steps. The QD is performed in the NVT ensemble (isochoric–isothermal conditions) using a Nosé thermostat^43^ for 5 ns at 500 K. 100 energy-minimized frames are obtained, and the frame with the lowest energy is taken as the starting structure for glass transition analysis. This procedure is commonly used for model preparation.^15,16,18^

### Glass Transition Analysis

An automated equilibration procedure specifically designed for glass transition analyses was developed. The routine consists of several *NpT* MD simulations (isobaric–isothermal conditions) using Berendsen^44^ pressure control (*p* = 1 bar) and Nosé thermostat, starting at 700 K. Each MD simulation runs over 500 ps, and the density is calculated every 5 ps and averaged over 125 ps. If the density difference does not exceed a certain threshold, then an additional MD simulation is conducted for data production. Similarly, if during data production the density differences do not exceed the threshold, then the trajectory is saved, and the program proceeds to the next temperature step. If at any point during the MD simulation the density difference exceeds the specified threshold, the MD simulation is continued until the value falls below the limit. This routine allows careful structure equilibration, and artificial inconsistencies are largely avoided. After the first temperature of 700 K, the routine carries out simulations in the temperature range from 600 K to 200 K in 50 K steps. One last *T*-step is performed at 100 K This procedure is also applied for small models but is terminated at 450 K to obtain equilibrated start structures for the poling simulations.

Density and temperature values of the different *T*-steps are saved during the routine. Subsequently, the glass transition (*T*_g_) is determined using the density–temperature plot. Two linear regressions are calculated, corresponding to the molten and glassy states of the polymer. The 11 data points resulting from the above-described procedure allow different combinations of regressions to be combined (e.g., 7 + 4, 6 + 5, ..., 4 + 7). The combination of regressions with the largest weighted average correlation coefficient is determined by using a Python script.

Experimental values are measured by differential scanning calorimetry (Mettler Toledo, DSC 1 STAR^e^ System) in three cycles, with each cycle ranging from 293 K to 523 K and 523 K to 293 K, at a heating or cooling rate of Δ*T* = 10 K min^–1^, respectively. The first heating cycle is for the equilibration of the system. The glass transition values of the second and third heating cycles are averaged, leading to the final value.

### Order Parameter, Poling, and Relaxation

The order parameter ⟨cos^3^ *θ*⟩ is commonly used to describe the alignment of a collection of dipoles and is of great importance for the estimation of electro-optic activity.^45^ The angle between one dipole and the direction of the applied electric field is given by the angle *θ*. Before MD methods were readily available and computationally feasible, theoretical models were developed to calculate the order parameter, e.g., for an ideal gas (rigid, noninteracting spheres). Within this model, the order parameter is described using the Langevin function

1 $$\left\langle \cos^{3}\theta \right\rangle =L_{3}\left(x\right)=\left(1+\frac{6}{x^{2}}\right)\coth x-\frac{3}{x}\left(1+\frac{2}{x^{2}}\right)$$

with

2 $$x=\frac{\mu E}{kT}$$

where *μ* is the molecular dipole moment, *E* is the applied electric field, *k* is Boltzmann’s constant, and *T* is the absolute temperature.^15,46^ In our MD approach, the order parameter ⟨cos^3^ *θ*⟩ is calculated frame by frame. At the end of the poling and relaxation MD simulations, the order parameter ⟨cos^3^ *θ*⟩ is averaged over the last 2 ns.

In our poling and relaxation procedure, the electric field is applied in the *z*-direction in an *NpT* ensemble with a Nosé thermostat and Berendsen pressure control. The protocol consists of three simulation stages:

(1) Poling above *T*_g_ for 20 ns
(2) Poling at room temperature for 10 ns (far below *T*_g_)
(3) Relaxation at elevated temperature (but well below *T*_g_) for 20 ns

The first two steps are experimentally well-established and commonly applied. First, chromophore reorientation should be facilitated at elevated temperatures above the glass transition. The second simulation stage serves to conserve the chromophore alignment in the vitrified polymer host. The last simulation stage was introduced to trigger reorientation and investigate the long-term stability of the chromophore orientation. In this context, poling efficiency and alignment stability are introduced, referring to the order parameter after the second and third simulation stages, respectively.

Different electric field strengths are applied, ranging from 0.5 to 5 kV *μ*m^–1^. A field strength of 5 kV *μ*m^–1^ is chosen for the majority of simulations. This field strength is stronger than that applied in experimental setups (0.1 kV *μ*m^–1^ to 0.2 kV *μ*m^–1^),^47,48^ but necessary to achieve equilibration on the time scale of simulations. This dilemma is commonly known in molecular modeling. Kim and Hayden^15^ had to apply much stronger fields in their simulations (up to 50 kV *μ*m^–1^), but with increased computing capabilities over the last few years, the applied field strengths were reduced, and simulation time could be maximized to produce meaningful results, i.e., steady-state/fully equilibrated structures.^16–19^

### Optical Activity Estimation

Macroscopic optical activity is described by the electro-optical (EO) tensor element *r*_333_. The first two indices are usually contracted for symmetry reasons (*r*_*ijk*_ = *r*_*jk*_ = *r*_*ik*_), so that *r*_333_ = *r*_33_.^47^ The EO coefficient *r*_33_ is related to the macroscopic second-order susceptibility $\chi_{\mathrm{zzz}}^{\left(2\right)}$ and the microscopic, molecular hyperpolarizability *β* by^47,49^

3 $$r_{33}=\frac{2\chi_{\mathrm{zzz}}^{\left(2\right)}}{n_{z}^{4}}=\frac{2N_{c}\beta f_{0}f_{\lambda }^{2}\left\langle \cos^{3}\theta \right\rangle }{n_{z}^{4}}$$

where *N*_c_ is the number density of chromophores, *f*_0_ and *f*_*λ*_ are local field factors (LFFs), ⟨cos^3^ *θ*⟩ is the order parameter of the chromophores, and *n*_*z*_ is the refractive index with respect to the polar axis. The Onsager LFF *f*_0_ relates the local electric field to the applied electric field, and the Lorenz–Lorentz LFF *f*_*λ*_ relates the local optical field to the external optical field (more details are given in Section S5).^47^

The refractive index in *z*-direction of the host–guest model is given by

4 $$n_{z}=\sqrt{\varepsilon_{\lambda }+4\pi N_{c}\left(\alpha_{c,||}-\alpha_{c,⊥}\right)\left[\left\langle \cos^{2}\theta \right\rangle -\frac{1}{3}\right]}$$

where *ε*_*λ*_ is the permittivity of the host–guest model at the wavelength of the EO experiment *λ*, *α*_c,||_ and *α*_c,⊥_ are the chromophore polarizabilities parallel and perpendicular to the dipole moment, and ⟨cos^2^ *θ*⟩ is another order parameter. The equation was taken from ref 18 and combined with an equation of ref 50 as outlined in eqs S15–S24.

In this study, we adopt the method developed by Tu et al.^18^ and Zhang et al.^19^ Additionally, we include PCM calculations to avoid local field factors *f*_0_ and *f*_*λ*_. The results of both methodologies are compared to experimental measurements. The full derivation and all necessary equations are given in Sections S5–S8 for better comparison.

## Results and Discussion

### Glass Transition Analysis

The glass transition (*T*_g_) analysis was applied to large models. The packing process of the large host–guest systems with more than 30 chromophores was challenging and had to be repeated in a few cases to achieve successful completion of the Monte Carlo task in the Amorphous Cell module. To avoid this time-consuming step at the beginning of the host–guest modeling, we decided to create only single models of each concentration for the *T*_g_ analysis, but carried out four equilibration MD simulations to explore the reproducibility of the *T*_g_ results. The Monte Carlo packing task for large pure PMMA models was more robust and less prone to fail, so multiple different large PMMA models were generated. The results of the *T*_g_ analysis for this larger set of models are attached in Figure S1.

The *T*_g_ is determined in order to derive further meaningful simulation parameters, i.e., the poling temperature (above *T*_g_) or the relaxation temperature (close to, but below *T*_g_). Figure 2 illustrates an example of the density–temperature (*ρ*/*T*) relationship for a pure PMMA model.

**Figure 2:**
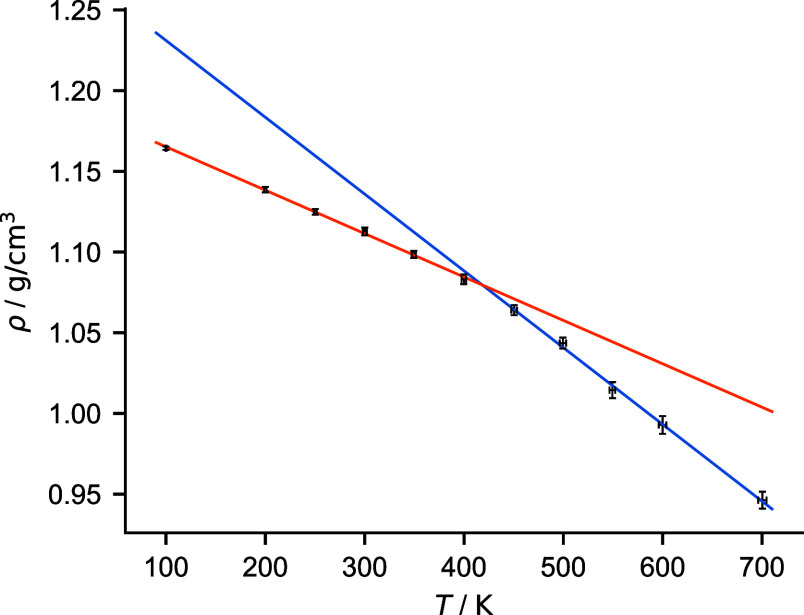
Example of a glass transition analysis graph. The intersection (estimated glass transition, here: *T*_g_ = 418 ± 11 K) is calculated based on the pair of linear regressions with the highest average correlation coefficient (here: *R* ^2^ = 0.9985).

As outlined above, different sets of data points may be chosen for linear regression analysis. The pair of linear regressions with the highest average correlation coefficient (*R* ^2^) is chosen for the determination of the intersection (*T*_g_). Figure 3 shows estimated glass transitions for PMMA with different amounts of chromophore C3 compared to experimental values. Each *T*_g_ value displayed in Figure 3 is an average of four equilibration MD simulations, and the error bars represent the standard deviation. For experimental values, error bars are omitted due to deviations being less than two Kelvins.

**Figure 3:**
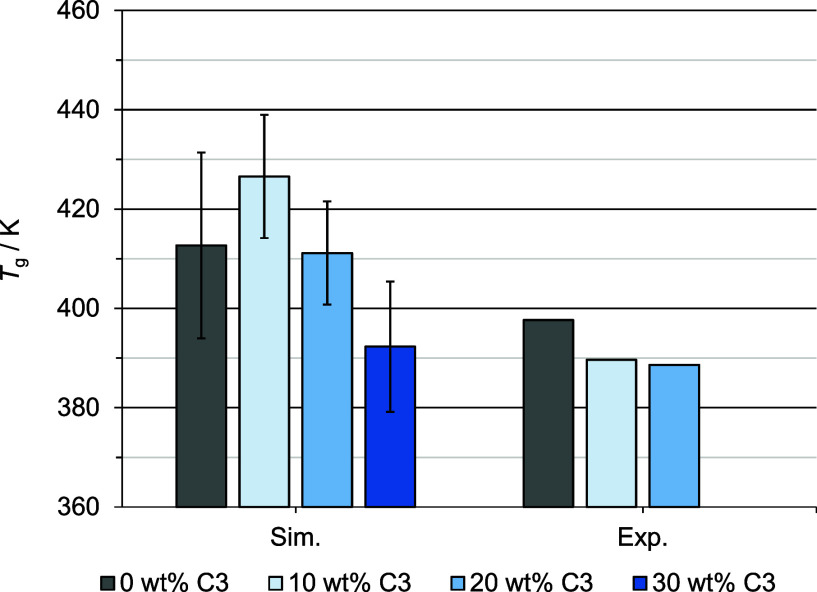
Glass transitions estimated from MD simulations (sim.) versus experimental values (exp.) depending on chromophore loading. The 30 wt% model is hypothetical and thus only available in simulation.

The glass transition temperatures of the simulation models are overestimated compared to the experimental values. The overestimation for the host–guest models is larger than that for the pure polymer but decreases with increasing chromophore content. The overestimation (e.g., 20 K to 100 K)^16,18,51–53^ is a commonly known effect arising from larger cooling rates compared to the experiment (Δ*T*_exp_ = 10 K min^–1^ vs Δ*T*_sim_ = 10 K ns^–1^).^18,53^

The modeling strategy chosen aims at the simulation of large polymer structures and a variety of independent models. The equilibration script introduced in the computational methods section was developed to reduce the aforementioned overestimation and to obtain results that are as comparable as possible to the experimental data. Compared to the results of previously conducted *T*_g_ analyses of pure PMMA^51,52,54,55^ or *T*_g_ analyses of PMMA host–guest materials,^16–18^ very good agreement with experimental values was reached for large models by thorough equilibration and usage of a well-suited force field (COMPASS III).

### Order Parameter, Poling, and Relaxation

Figure 4 shows an example of the progression of the order parameter of a host–guest model during and after the standard poling and relaxation routine.

**Figure 4:**
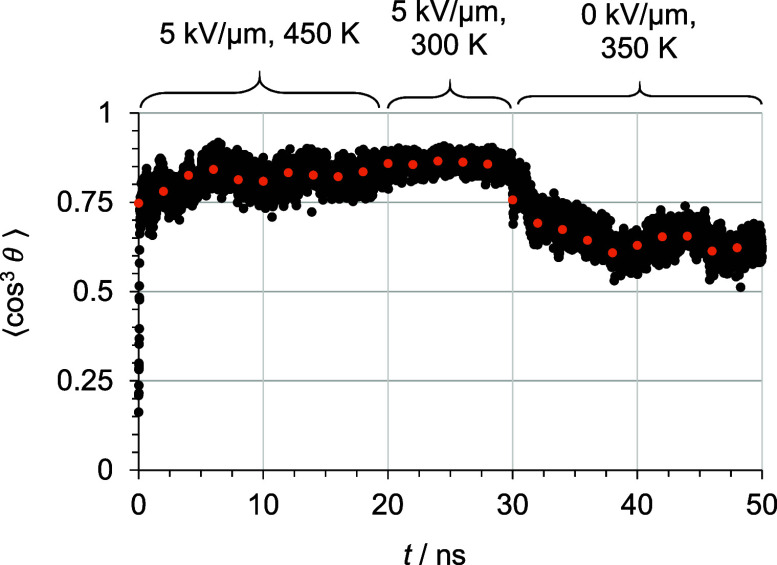
Order parameter ⟨cos^3^ *θ*⟩ during and after the standard electric-field poling process of one exemplary structure (10 wt% C3 in PMMA, black bullets: order parameter of every single frame, orange bullets: 2 ns averages). The average value at 28 ns is referred to as poling efficiency, and the last average value at 48 ns is referred to as alignment stability.

Black bullets represent the framewise calculated order parameter, and orange bullets represent averages over 2 ns. The average value at 28 ns (last of the poling stage) is referred to as poling efficiency, and the average value at 48 ns (last of the relaxation stage) denotes the alignment stability. The first simulation stage (0 ns to 20 ns) focuses on achieving the highest possible order parameters at temperatures above *T*_g_ using field strengths close to experimentally accessible ones. These were chosen to reach a steady state within a reasonable MD time frame (see computational methods section: Order Parameter, Poling, and Relaxation). The second simulation stage (20–30 ns) is carried out at room temperature with the same applied electric field. The polymer is vitrified, freezing molecular mobility and preserving chromophore alignment. In order to relax nonequilibrium arrangements due to the strong electric field and to account for local interactions, a simulation is conducted at slightly elevated temperatures (below *T*_g_) without an electric field. This simulation, performed from 30 ns to 50 ns, aims to achieve a chromophore alignment comparable to the experiment.

We aim to determine and verify parameters for a reliable electric field poling process that provides meaningful results compared with the theoretical model and expected experimental behavior. The poling efficiency value is regarded as a theoretical maximum value and may not be directly compared to experimental data. In contrast, the alignment stability values can be used for comparison with experimental data.

Figure 5 shows the poling efficiency and alignment stability of different host–guest models with various electric field strengths applied.

**Figure 5:**
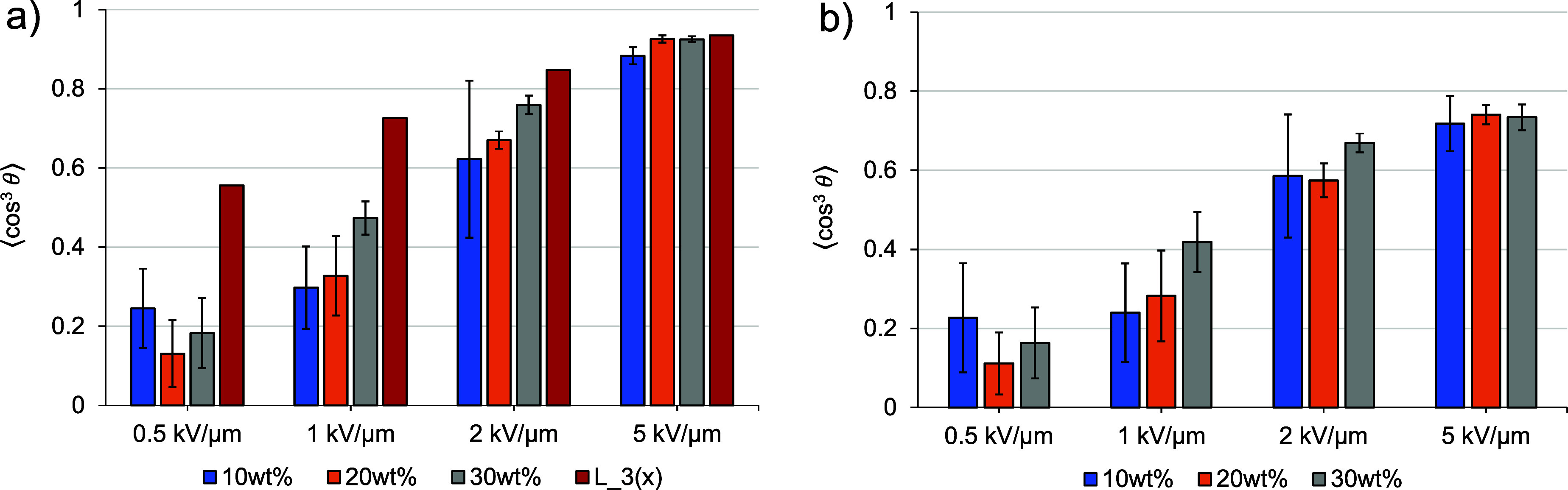
(a) Poling efficiency while applying different field strengths and (b) alignment stability after the application of different electric fields in models with different weight percentages of C3 in PMMA (each pillar represents the average of three independent models; error bars represent the standard deviation). Red pillars representing values of the third Langevin function (*L*_3_(*μ*E_p_/*kT*)), the upper limit of the order parameter for a noninteracting rigid gas model.

Electric field strengths were initially chosen close to experimentally used ones (0.5 to 2 kV *μ*m^–1^). Each pillar represents the average of three independent poling and relaxation MD calculations. Red pillars show results for the theoretical noninteracting rigid gas model (see eq 1), where *L*_3_ = **μ*E*_p_/*kT* , with the corresponding electric field strengths *E*_p_ of 0.5; 1; 2; and 5 kV *μ*m^–1^). The noninteracting rigid gas model shows good agreement with simulation results, as reported in previous investigations^15–17^ and serves as a reference. It was observed that the noninteracting rigid gas model is applicable to host–guest systems with low chromophore concentrations or in cases with large applied electric fields. The MD calculations presented herein show smaller order parameter values than the noninteracting rigid gas model at all poling field strengths. The order parameters resulting from electric field poling at electric field strengths of 0.5 and 1 kV *μ*m^–1^ show a low standard deviation and are 30–40% below the values for the rigid gas model. This result implies that the chromophores may not be thoroughly equilibrated until reaching a steady state, as the chosen simulation time of 20 ns was presumably too short. A similar picture is observed in the individual order parameter diagrams (shown in Figure S2).

An electric field poling with 2 kV *μ*m^–1^ shows a significant improvement and only underestimates the values of the noninteracting rigid gas model by less than 20%. This poling strength may be suitable for further MD calculations, but larger uncertainties are observed for low-concentration models. Electric field poling with 5 kV *μ*m^–1^ provides a poling efficiency comparable to the noninteracting rigid gas model, implying that local interacting forces are less dominant during poling than with lower electric field strengths. The alignment stability obtained from simulations with 5 kV *μ*m^–1^ is approximately 20% lower than the value obtained by the noninteracting rigid gas model. The lower order parameter value after relaxation may be due to local interactions of chromophores in the host matrix. The alignment stability for the models poled with lower electric fields does not show significant smaller values than for the poling efficiency, which may imply incomplete alignment due to insufficient simulation time during poling. In order to achieve a steady state and good reproducibility regardless of concentration, an electric field of 5 kV *μ*m^–1^ was chosen for the standard procedure.

The poling efficiency values for 2 and 5 kV *μ*m^–1^ poled models are in quite good agreement with the noninteracting rigid gas model. The alignment stability values are notably lower than the values obtained with the noninteracting rigid gas model (or poling efficiency values), especially for lower electric fields. Due to the large deviation of the chromophores presented in this work from an ideal spherical shape, a larger discrepancy between the noninteracting rigid gas model and the MD result was expected and finally observed. The prolate spheroid shape promotes alignment stability by decreasing the tendency of reorientation in contrast to a sphere but requires stronger electric fields to reach equilibrium alignment. This relationship is in accordance with the work of Makowska-Janusik et al.,^16^ who investigated the influence of different chromophore shapes on poling efficiency and alignment stability. Hence, the noninteracting rigid gas model is not applicable in general for typical experimental conditions, as also concluded earlier in ref 17.

As a result of the analyses mentioned above, all simulations were generally carried out with a field strength of 5 kV *μ*m^–1^. This is up to 1 order of magnitude larger as the poling field of Tu et al.,^18^ who investigated a Disperse Red chromophore derivative (DRD) in PMMA. As shown above, this stronger field strength is necessary in order to align and equilibrate the significantly heavier and sterically more demanding chromophore C3 in the polymer matrix (compare molar masses: *M*(C3) ≈ 1.5 *M*(DRD)). The decision for a stronger electric field is based on a compromise between the calculation time (*t*_CPU_) and the appropriate equilibration time (*t*_MD_). In this context, a total simulation time of 50 ns for poling and relaxation was considered appropriate.

With the previously validated settings, we performed poling and relaxation simulations. The obtained poling efficiency and alignment stability values are summarized in Figure 6. With increasing chromophore content, the poling efficiency increases slightly but not significantly. The alignment stability shows a trend similar to that of the poling efficiency. Overall, we find an average order parameter of at least 0.7, independent of the chromophore concentration.

**Figure 6:**
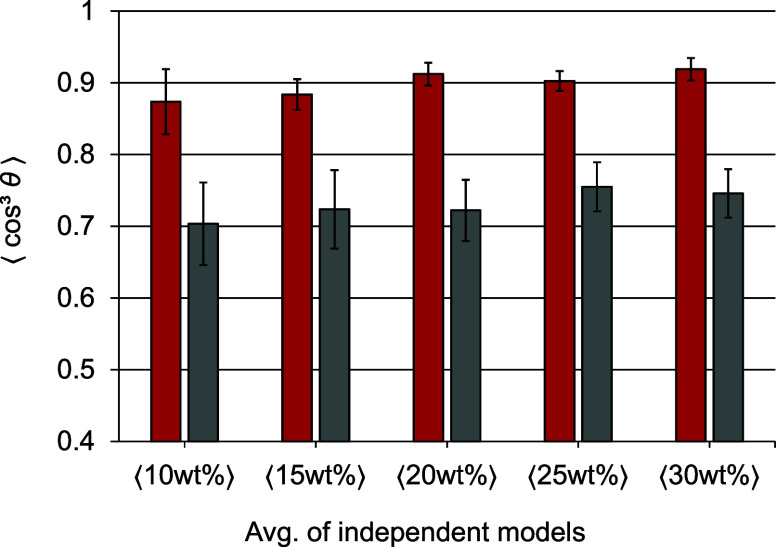
Poling efficiency (red bars) and alignment stability (gray bars) after the application of an electric field of 5 kV *μ*m^–1^ on models with different weight percentages of C3 in PMMA (⟨*x*⟩: average of nine independent models; error bars representing the standard deviation).

Four extended relaxation simulations were carried out to investigate the long-term and high-temperature stability. For both the highest and lowest chromophore concentrations, a host–guest model was selected based on the median order parameter value after the standard poling and relaxation program. The two previous relaxation MD steps of 20 ns were extended to 100 ns to demonstrate the stability of chromophore alignment over longer time scales. Additionally, two more MD simulations were initiated at 450 K (above *T*_g_) after room-temperature poling to emphasize the significance of vitrification. Figure 7 shows the relaxation behaviors of the four different simulation experiments.

**Figure 7:**
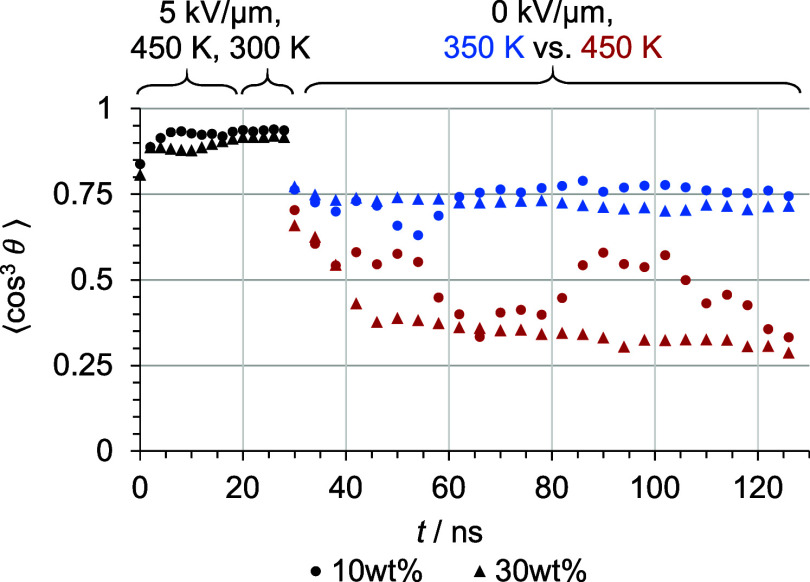
Order parameter ⟨cos^3^ *θ*⟩ during long-term relaxation simulations at temperatures below (blue) and above (red) the estimated glass transition with the lowest (dots) and highest (triangles) weight percentages of C3 in PMMA.

At temperatures below *T*_g_, the chromophore alignment is preserved and comparable to the short standard relaxation MD simulation of 20 ns, supporting the expediency of the general material concept and simulation protocol. The order parameter for the models simulated at temperatures above *T*_g_ decreases to a significantly lower value for both the high and low concentration models. The order parameter for the low concentration model fluctuates in the range of 0.3–0.6. For the high concentration model, the order parameter slowly decreases to 0.3 without notable fluctuations.

Figure 8 shows the different phase behaviors of the chromophores. In the whole simulation, the low-concentration model consists of one aggregate and a few isolated or weakly associated chromophores. The aggregate, with four out of seven chromophores, stays aligned, whereas the three isolated or weakly associated chromophores can easily reorient, resulting in a fluctuating order parameter. At high chromophore concentrations, the chromophores realign collectively, and the overall order parameter decreases more steadily (c.f. Figure 7).

**Figure 8:**
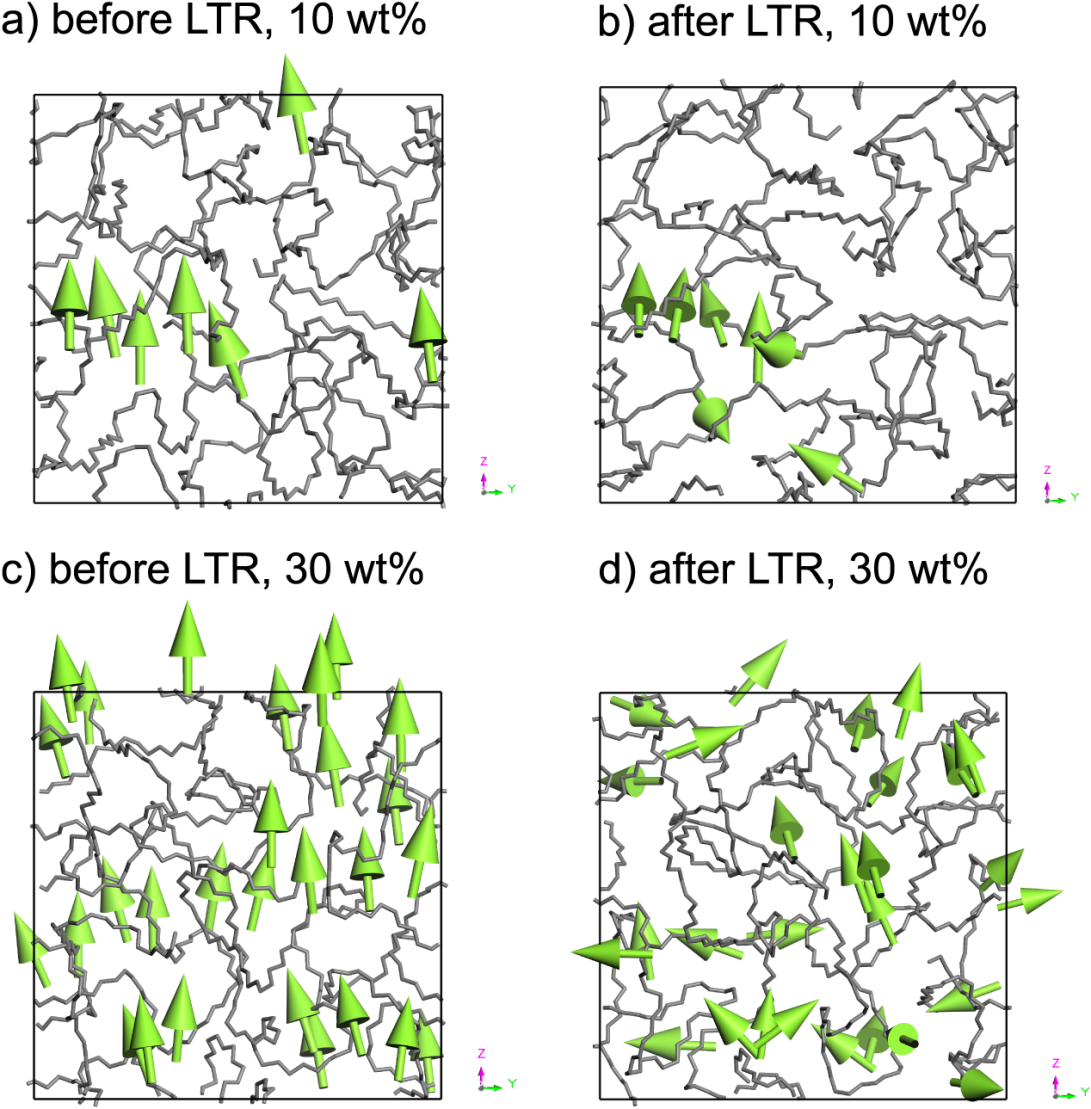
Phase behavior of chromophores in a polymer matrix at low and high concentrations in the course of long-term relaxation (LTR) at a temperature above *T*_g_. Figure (a) shows a model containing 10 wt% C3 before the LTR step, and Figure (b) shows the model after LTR. Figures (c, d) illustrate the LTR for a 30 wt% model.

### Optical Activity Estimation

We next applied the obtained orientation parameters to calculate experimentally accessible *r*_33_ values. For this, Onsager local field factors (LFFs) *f*_0_ are calculated using experimental values obtained from the literature and the well-established equation (eq S6). It has to be emphasized that the equation derived by Onsager is a simplified model to relate gas-phase computations and solution-phase measurements.^56^ A more rigorous quantum mechanical treatment can circumvent the underlying model assumptions (e.g., a spherical cavity as a reaction field). For a better representation of the local field effects, there are some strategies shown to take cavities with proper molecular shape or solvent–solute interactions into account.^56,57^ However, the rigorous estimation of the local field factor is beyond the scope of this article and is therefore omitted. Instead, it is suggested to include polarizable continuum models (PCM) in DFT calculations of the (hyper)­polarizability to account for local field effects, presumably making them and their sophisticated estimation procedure obsolete, especially for treatment in a polymer matrix.

Table 2 presents the polarizability and hyperpolarizability values for the chromophore guest molecule C3.

**Table 2:** Elements of the Polarizability Tensor **α** and the Total Hyperpolarizability *β*_tot_ at a Wavelength of λ = 970 nm in the T-Convention^a^ for the C3 Chromophore (Further Values in Table S2)

	Polarizability^b^			Hyperpolarizability^c^
	*α*_*xx*_	*α*_*yy*_	*α*_*zz*_	*β*_tot_
*in vacuo*	38.4	58.8	162.3	490.1
PCM	43.4	66.3	240.2	1068.6

a Ref 58: origin and meaning of different well-established conventions.

b In 10^–24^ esu.

c In 10^–30^ esu.

Polarizabilities are calculated *in vacuo* and with a polarizable continuum model (PCM) using DFT methods (see Computational Methods), representing a fast and reliable approach. The *in vacuo* values might underestimate the polarizability in a polymer matrix because the gas-phase (*in vacuo*) calculations do not account for interactions with a surrounding medium.^47^ Calculations with the MP2 method or even more elaborate methods, e.g., coupled cluster methods, might be in better agreement with experimental values^41,59^ but require exceedingly large computational effort for the large chromophores of interest. The simulation strategy presented here balances computational effort with accuracy of results to provide meaningful estimates. To validate this approach, we contextualized the obtained values with experimental values of comparable chromophore molecules. The main objective is not to compare absolute values but rather highlight the applicability of the herein-devised theoretical characterization method for providing suitable and comparable estimates to the experiment.

Quilty^60^ presented an overview of recently developed chromophore molecules that are of interest to materials scientists today. The molecular performance is expressed in terms of the experimentally determined hyperpolarizability *β*_*zzz*_, which is in the range of 300 × 10^–30^ esu to 1200 × 10^30^ esu. Often, *β* is assumed to be *β*_*zzz*_, i.e., in most cases the largest tensor component (*β* = *β*_tot_ ≈ *β*_*zzz*_),^1,46,61^ because the experimentally measured macroscopic tensor **β** can be quite complex, and a reasonable and practical simplification can be made when only one component needs to be considered.^46^ To ensure full comparability, additional *β* values of the C3 chromophore molecule investigated in this paper are outlined in the Table S2, e.g., static and wavelength-dependent *β*_*zzz*_ values. The aforementioned approximation *β*_*zzz*_ ≈ *β*_tot_ also applies to our theoretical investigations (e.g., $\beta_{zzz}^{\mathrm{PCM}}=990\times 10^{-30}\mathrm{esu}\approx 1070\times 10^{-30}\mathrm{esu}=\beta_{\mathrm{tot}}^{\mathrm{PCM}}$ ) and the theoretically obtained hyperpolarizability *β*_*zzz*_ is in the range with experimentally observed values of contemporary chromophores.^60^

Suponitsky et al.^59^ found the error in theoretically determined *β* values to be in the range of 15–45% for donor-/acceptor-substituted benzenes and stilbenes, if solvent and frequency effects are not considered. This error was assessed to be comparable to the uncertainty in the experimental data. In the present study, the chromophore “C3”, with a more pronounced donor/acceptor electron structure, was investigated, and a strong solvent and frequency dependency is present. Each contribution (either solvent or frequency dependencies) may increase the hyperpolarizability value compared to the static, *in vacuo* value by a factor of approximately two. In experimental investigations, there is no direct measurement technique for the determination of the Pockels effect *β*(−*ω*;0,*ω*) for chromophores in host–guest polymer systems^41,60^ so that measurements in solution (e.g., Hyper-Rayleigh scattering (HRS) or electric-field-induced second harmonic generation (EFISHG)) are necessary, and the experimentally observed uncertainty in *β*(−2*ω*;*ω*,*ω*) is strongly dependent on the solvent polarity.^41,60^ In conclusion, it is recommended that solvent effects and frequency dependencies should be considered for “large” / multi-functional chromophore molecules. Thus, there is good evidence that the (hyper)­polarizabilities obtained by PCM are reliable and account for solvation effects.

Table 3 summarizes all necessary values for the calculation of the *r*_33_ value.

**Table 3:** Summary of Data for PMMA/C3 Host–Guest Modeling^a^

Model set	*ρ*/gcm^–3^	*N*_c_/10^20^cm^–3^	⟨cos^2^ *θ*⟩	⟨cos^3^ *θ*⟩	*ε*_λ_	$$\varepsilon_{\lambda }^{\mathrm{PCM}}$$	*n*_*z*_	$$n_{z}^{\mathrm{PCM}}$$	*r*_33_/pm V^–1^	$r_{33}^{\mathrm{PCM}}$ /pm V^–1^
0 wt%	1.117 ± 0.003	0.0	–	–	2.18	–	1.48	–	–	–
After poling										
10 wt%	1.128 ± 0.004	1.4	0.91 ± 0.04	0.87 ± 0.05	2.30	2.29	1.57	1.57	27.8 ± 1.7	18.2 ± 1.2
15 wt%	1.132 ± 0.006	2.3	0.92 ± 0.02	0.88 ± 0.02	2.38	2.35	1.63	1.63	40.4 ± 1.2	25.7 ± 0.8
20 wt%	1.134 ± 0.006	2.9	0.94 ± 0.01	0.91 ± 0.02	2.44	2.39	1.68	1.68	48.7 ± 1.1	30.3 ± 0.7
25 wt%	1.136 ± 0.004	3.8	0.93 ± 0.01	0.90 ± 0.01	2.51	2.45	1.73	1.72	56.3 ± 1.2	34.3 ± 0.7
30 wt%	1.138 ± 0.004	4.5	0.94 ± 0.01	0.92 ± 0.02	2.59	2.50	1.79	1.77	62.5 ± 1.5	37.4 ± 0.9
After relaxation										
10 wt%	1.108 ± 0.006	1.4	0.77 ± 0.05	0.70 ± 0.06	2.28	2.26	1.55	1.55	22.9 ± 2.2	15.3 ± 1.5
15 wt%	1.110 ± 0.006	2.3	0.79 ± 0.05	0.72 ± 0.05	2.35	2.32	1.60	1.60	34.4 ± 3.2	22.2 ± 2.1
20 wt%	1.111 ± 0.004	2.9	0.79 ± 0.03	0.72 ± 0.04	2.40	2.37	1.64	1.64	40.9 ± 3.0	25.9 ± 2.0
25 wt%	1.112 ± 0.004	3.7	0.82 ± 0.03	0.75 ± 0.03	2.48	2.42	1.69	1.68	49.9 ± 3.0	30.9 ± 1.9
30 wt%	1.113 ± 0.004	4.4	0.81 ± 0.03	0.75 ± 0.03	2.55	2.47	1.73	1.72	54.9 ± 3.5	33.5 ± 2.2

a The model set is described by the rounded weight percentage of chromophore C3 incorporated into the PMMA polymer host. All values are average values of nine independent models and their corresponding standard deviations. The permittivity *ε_λ_* is estimated for a wavelength of *λ* = 970 nm. Local field factors are replaced by polarizabilities from polarizable continuum model (PCM) calculations for “PCM”-denoted values.

The weight percentages in the model set column are approximate values and serve as labels for the model set. Each model set consists of nine independent structures, as given in Table 1. The second column shows the average density of all nine models at each concentration for the last 2 ns of the trajectories. The number density of chromophores *N*_c_ is calculated based on the average model set density, *ρ*. The order parameter values ⟨cos^2^ *θ*⟩ and ⟨cos^3^ *θ*⟩ are average values of the last 2 ns of each trajectory section (either poling or relaxation) and are also averaged over the nine independent models of each set. The permittivity at the wavelength of the EO experiment *ε*_*λ*_ and the refractive index in *z*-direction *n*_*z*_ are calculated as outlined in eqs S13, S21, and S23. Finally, the electro-optic activity, expressed as the *r*_33_ value, is calculated with eq 3 and represents an average value for the respective model set. PCM values are calculated using (hyper)­polarizabilities obtained from PCM calculations. In return, local field factors are obsolete for these calculations and therefore are omitted (compare eqs S5–S24). The main uncertainty in the electro-optic tensor element, Δ*r*_33_ (2–10%), arises from the uncertainty in the order parameters (Δ⟨cos^2^ *θ*⟩, Δ⟨cos^3^ *θ*⟩: 1–6%). The uncertainties in the number density of chromophores Δ*N*_c_ (<0.1%), permittivity Δ*ε*_*λ*_ (<0.1%), and refractive index Δ*n*_*z*_ (<0.5%) are omitted.

In Figure 9, the *r*_33_ values (black: values after traditional LFF approximation/red: newly introduced PCM-calculated results) are plotted against the average chromophore number density *N*_c_.

**Figure 9:**
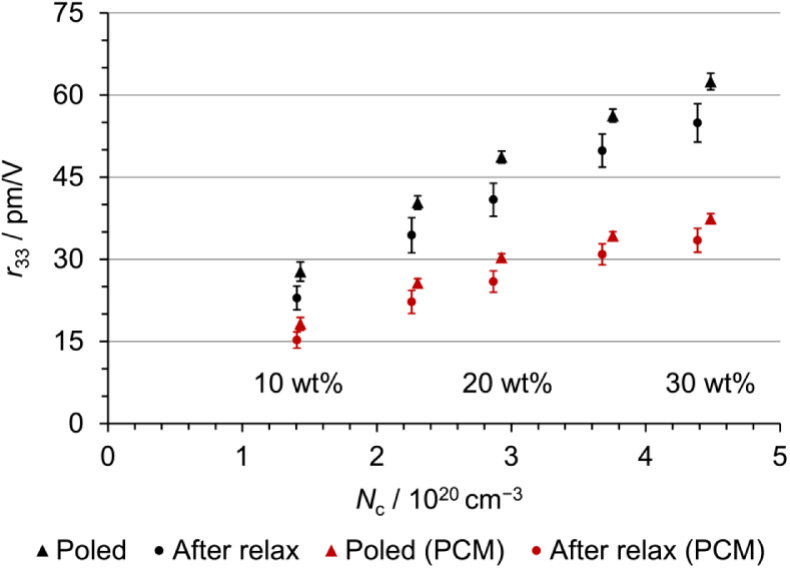
Electro-optic (EO) activity expressed in terms of the estimated electro-optic tensor element *r*_33_. The term “poled” refers to the EO activity directly after the poling process (ideal maximum order parameter value), and “after relax” refers to the EO activity in the application case (slightly decreased order parameter value because of elevated temperature and the occurrence of some reorientation). The local field approximated values are represented by black symbols, and values based on PCM calculations are represented by red symbols.

An increase in the EO effect can be observed with increasing chromophore content. A linear trend is not observed at high chromophore concentrations, as the refractive index of the host–guest material *n*_*z*_ contributes disproportionately. The nonlinearity is even more strongly pronounced for the PCM-based estimations because the PCM correction influences the polarizability more significantly than the hyperpolarizability. Therefore, the correction affects the refractive index to a greater extent, and the EO activity is more attenuated at higher chromophore concentrations. At concentrations above 15 wt% C3 in PMMA (or for the PCM-based estimations above 20 wt%), EO activities larger than 30 pm V^–1^ are achieved, a value commonly known for the traditional crystalline lithium niobate (LiNbO_3_). In experiments, polymeric thin films of PMMA could be loaded with up to 20 wt% C3 before aggregation occurred.

Table 4 shows refractive index (RI) values determined using ellipsometry measurements in comparison to theoretically estimated values (using the LFF-based approach and PCM-based calculations).

**Table 4:** Refractive Indices of Nonpoled PMMA/C3 Host–Guest Materials^a^

wt% C3	Exp.	LFF	PCM
0	1.483^b^	1.477	1.477
15	1.595^c^	1.532	1.524

a Ellipsometry measurements (Exp.) compared to estimations with the local field factor (LFF) approach and the polarizable continuum model (PCM) method.

b On glass.

c On Si wafer.

The values determined using ellipsometry are the averages of three samples. More information about the device and optical models of the experimental measurement is given in Figure S3. The experimental values are in good agreement with the theoretical estimates and are remarkably independent of the chosen approach (LFF or PCM).

The highest $r_{33}^{970 nm}$ value observed in a Teng–Man measurement setup^62^ was (20.9 ± 5.1) pm V^–1^ for a thin-film sample of 15 wt% C3 in PMMA. The sample preparation, poling procedure, Teng–Man measurement setup, and error estimation^63,64^ are supplied within Section S9. The experimental value is in good agreement with the value estimated using the PCM approach $(r_{33}^{\mathrm{PCM}}$ = (22.2 ± 2.1) pm V^–1^), whereas the LFF approach overestimates the EO coefficient (*r*_33_ = (34.4 ± 3.2) pm V^–1^). The simulation represents optimal EO activity values based on perfect alignment and a low degree of orientational relaxation, i.e., an upper possible limit for experimental values.

Table 5 relates the results of this study to previous MD studies. The *r*_33_ values of the previous MD studies^18,19^ are adapted to the models and assumptions made in this paper (for details, refer to eq S25).

**Table 5:** Comparison with Previous MD Studies (Values are Adapted from Refs 18, 19, © 2007 American Chemical Society)^a^

Modeled systems and parameters	*N*_c_/10^20^ cm^–3^	$\left\langle \cos^{3}\theta \right\rangle$	*r*_33_/pm V^–1^
C3 in PMMA, *E*_p_ = 5 kV *μ*m^–1^, *f*_0_ = 1.60, *β*_tot_ = 490.1	1.4	0.70 ± 0.06	22.9 ± 2.2
	2.3	0.72 ± 0.05	34.4 ± 3.2
	2.9	0.72 ± 0.04	40.9 ± 3.0
	3.7	0.75 ± 0.03	49.9 ± 3.0
	4.4	0.75 ± 0.03	54.9 ± 3.5
DRD in PMMA^18^, *E*_p_ = 0.5 kV *μ*m^–1^, *f*_0_ = 1.60, *β*_tot_ = 86.8	5.8	0.31 ± 0.36	7.7
	9.3	0.35 ± 0.37	12.5
	11.7	0.33 ± 0.36	14.4
Lemke chr. in APC^19^, *E*_p_ = 0.5 kV *μ*m^–1^, *f*_0_ = 1.57, *β*_tot_ = 111.7	2.0	0.34 ± 0.42	3.8
	3.6	0.36 ± 0.33	6.8
	6.0	0.39 ± 0.32	11.1

a The total hyperpolarizability *β*_tot_ is given in units of 10^–30^ esu, and the *r*_33_ values are adapted to the models and assumptions of this study (for details, please refer to eq S25). *E*_p_ denotes the poling field strength, and *f*_0_ is the Onsager local field factor, which depends on the polymer system (details are given in eq S6). APC: amorphous polycarbonate; chr.: chromophore.

The hyperpolarizability *β*_tot_ of the C3 chromophore is four to five times larger, allowing much lower chromophore concentrations to be incorporated and avoiding aggregates observed in experiments at C3 concentrations above 20 wt%. The order parameter values ⟨cos^3^ *θ*⟩ of previous studies^18,19^ are between 0.3 and 0.4, which is similar to the expected value calculated via the Langevin functions to describe the rigid ideal gas model. The theoretical order parameter for C3 in PMMA, calculated using the Langevin functions, is slightly larger (0.6–0.9 because of the larger dipole moment, lower temperature, and stronger electric field, compare Figure 5). The previous MD studies aimed at the application of an electric field strength comparable to the experiment. In this study, larger chromophore molecules were introduced, and the proper equilibration of the host–guest system was the focus. Even though the MD studies consisted of different poling parameters and chromophores, the obtained results are comparable. In both simulation cases, it was proven by the theoretically calculated order parameter values that the models are properly equilibrated under the applied field strengths. The smaller chromophores (DRD- and Lemke-type) can (re)­orientate more easily because of the smaller molecular mass and their smaller steric demand. However, it seems unlikely to yield larger order parameter values, as the smaller dipole moments of these molecules react less to the stronger electric fields. Taking this lower poling efficiency of the former host–guest model simulations into account, all the calculated *r*_33_ values are in overall accordance with the expectations, and the *r*_33_ values of the PMMA/C3 host–guest models are promising for future applications, possibly replacing the traditional crystalline lithium niobate.

Finally, the simulation results are set in relation to other published experimental results. Quilty^60^ examined different host–guest systems theoretically and compared the obtained results with experimental measurements. All theoretically determined maximum *r*_33_ values are in the same range as those presented here (6–51 pm V^–1^). The reference chromophore number density for all of these values was 1 × 10^20^cm^–3^. Both theoretical investigations show results in good accordance, but it is also stressed by Quilty^60^ that achievable *r*_33_ values are much lower in the experimental case (in the range of 2–14 pm V^–1^) than the calculated ones, because of aggregation lowering the extent of the optical effect. The presented *r*_33_ values for the C3 in PMMA host–guest systems here also indicate only optimal optical activities.

## Conclusion and Outlook

This paper highlights the prospects of modern polymer host–guest modeling. Several tools were devised, and the previously developed methodological framework of molecular dynamics (MD) techniques^15–19^ was revised and adapted to contemporary materials and the capabilities of computational resources currently available.

This study focused on the “C3” chromophore molecule as a promising candidate for host–guest materials today. The quantum mechanical characterization was extended to polarizable continuum model (PCM) calculations, implying a more straightforward estimation procedure for the optical activity (calculation of the electro-optic (EO) coefficient *r*_33_ without local field factors) and showing good agreement to experimental measurements and previous experimental and theoretical studies (regarding refractive index (RI), (hyper)­polarizability, and *r*_33_ values). In this study, a set of computationally feasible models and modeling parameters (size of polymer/host–guest models, time scales, and MD parameters) were developed and thoroughly validated, which may function as a reference for future computational investigations. An MD equilibration protocol was proposed to investigate phase transitions, e.g., the glass transition *T*_g_. This MD equilibration protocol is also applicable to equilibrate (anneal) models after building/packing (equilibration of start structures).

Another concern of this study was the theoretical elaboration of the EO coefficient *r*_33_ for the host–guest material (C3 in PMMA). Applying the methods devised by Tu et al.^18^ and Zhang et al.,^19^ some methodological steps were clarified, simplified, and further developed under the consideration of PCM calculations, giving an improvement compared to the previous method. The results are in good agreement with measurements and to already published experimental and theoretical results.

In a subsequent study, we will investigate the chromophore aggregation and phase behavior in detail. It will address questions concerning concentration limits for chromophores before aggregation inevitably occurs and how chromophores are distributed in the host polymer during the course of the applied simulation protocol. Another insightful objective of the following study is the analysis of aggregates in dependence on chromophore shape and concentration. With the published methods of this and the next study, the capabilities of molecular modeling techniques should be highlighted, which are paving the way for accelerated development of materials and may be supported in the future by the ever-increasing computational resources and methods (e.g., machine learning and quantum computing).

## Supplementary Material



## Notes

S1: Glass Transition Analysis; S2: Langevin Function and Order Parameter; S3: Electric Field Poling with Different Field Strengths; S4: Conventions and Units of the Hyperpolarizability; S5: Relation of Microscopic Properties to a Macroscopic Quantity, the Electro-optic Coefficient; S6: Refractive Index Estimation of a Host–Guest Model; S7: Optical Properties; S8: Relation to Previous Studies; S9: Sample Preparation, Poling Procedure, and Teng–Man Measurement; and S10: Ellipsometry Measurements (PDF)

## ⊥

Passed away on January 13, 2023.
