# Supplementary material for: Molecular Dynamics Simulations of Electric Field Poled Poly(methyl methacrylate) Doped with Tricyanopyrroline Chromophores
Source: J Phys Chem B. 2025 Jul 25;129(31):8015–27. doi: 10.1021/acs.jpcb.5c02832 (PMC12337094; doi:10.1021/acs.jpcb.5c02832)
Supplement: Supplementary file 1 [file jp5c02832_si_001.pdf]

Supporting Information: Molecular Dynamics Simulations of Electric Field Poled Poly(methyl methacrylate) Doped with Tricyanopyrroline Chromophores

Nils M. Denda,[†,‡] Erik Rohloff,[†] Florens R. Kurth,[¶,‡] Li Zhao,[¶,‡] Hans-Hermann Johannes,[¶,‡] Wolfgang Kowalsky,[¶,‡] Carolin König,[§,‡] Peter Behrens,[†,‡,‖] and Andreas M. Schneider[*,†,‡]

†*Institute of Inorganic Chemistry, Leibniz University Hannover, 30167 Hannover, Germany*

‡*Cluster of Excellence PhoenixD (Photonics, Optics, and Engineering – Innovation Across Disciplines), 30167 Hannover, Germany*

¶*Institute of High-Frequency Technology, Technische Universität Braunschweig, 38106 Braunschweig, Germany*

§*Institute of Physical Chemistry and Electrochemistry, Leibniz University Hannover, 30167 Hannover, Germany*

‖*Passed away on January 13, 2023*

E-mail: andreas.schneider@acb.uni-hannover.de

Phone: +49 (0) 511 762 3259. Fax: +49 (0) 511 762 3006

Contents

S1 Glass Transition Analysis S3

S2 Langevin Functions and Order Parameter S4

S3 Electric Field Poling with Different Field Strengths S4

S4 Conventions and Units of the Hyperpolarizability S6

S5 Relation of Microscopic Properties to a Macroscopic Quantity, the Electro-optic Coefficient r_{33} S7

S6 Refractive Index Estimation of a Host–Guest Model S10

S7 Optical Properties S12

S8 Relation to Previous Studies S14

S9 Sample Preparation, Poling Procedure, and Teng–Man Measurement S15

S10 Ellipsometry Measurements S17

References S19

S1 Glass Transition Analysis

As a supplement to the main manuscript, we provide the results of all glass transition analyses of the large PMMA model set. Figure S1 shows glass transition results for ten independently developed PMMA models, each consisting of 14 chains with 100 repeating units. The glass transition procedure and analysis is described in the computational

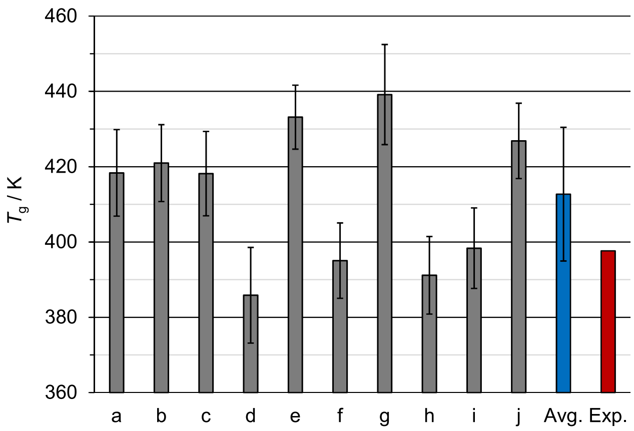

Figure S1: Glass transition estimated from molecular dynamics (MD) simulations of ten independent polymeric PMMA structures (a-j) compared to experimental values.

methods section of the main manuscript. The glass transition analysis for independent models shows an average value of (412.7 ± 18.7) K with a large standard deviation due to a high variance. The majority of simulated glass transitions show the expected (slight) overestimation of the T_g value compared to experimental measurements.

Glass transition analyses conducted on small models exhibited an even more pronounced variance and are therefore not presented herein. Different process parameters were tested, i. e. different temperature ranges and step sizes, as well as different equilibration times. It was recognized that the inclusion of smaller and numerous temperature steps as well as an increase in equilibration time led to smaller deviations and more reproducible results. All of these parameters had to be balanced with the computational effort in order to achieve a compromise between production time and the availability of a solid and substantiated data set.

S2 Langevin Functions and Order Parameter

The general definition of the Langevin function

$$L_n(x) \equiv \langle \cos^n \theta \rangle = \frac{\int_0^\pi \exp(-x\cos\theta)\cos^n\theta\sin\theta \mathrm{d}\theta}{\int_0^\pi \exp(-x\cos\theta)\sin\theta \mathrm{d}\theta} \quad \text{with} \quad x = \frac{\mu E}{kT} \tag{S1}$$

is taken from Ref S1, where μ is the molecular dipole moment, E is the applied poling field, k is Boltzmann's constant and T is the absolute temperature. Using this definition, it is possible to calculate different order parameters

$$\left.\begin{aligned} L_1(x) &= \coth(x) - \frac{1}{x} && = \langle \cos\theta \rangle \\ L_2(x) &= 1 + \frac{2}{x^2} - \frac{2}{x}\coth(x) && = \langle \cos^2\theta \rangle \\ L_3(x) &= \left(1 + \frac{6}{x^2}\right)\left(\coth(x) - \frac{1}{x}\right) - \frac{2}{x} && = \langle \cos^3\theta \rangle \end{aligned}\right\} \text{where} \quad x = \frac{\mu E}{kT} \tag{S2}$$

of an ideal rigid gas model (non-interacting hard spheres). The angle θ, i. e. the angle between one dipole moment and the applied electric field vector, can be measured using the trajectories of molecular dynamics (MD) simulations. The different order parameters, i. e. $\langle \cos\theta \rangle / \langle \cos^2\theta \rangle / \langle \cos^3\theta \rangle$ can be calculated frame by frame as ensemble averages. These values implicitly describe the interactions between the chromophores and the host polymer or other guest molecules.

S3 Electric Field Poling with Different Field Strengths

Figure S2 shows the individual order parameter evolution under different applied electric field strengths ranging from 0.5 kV μm^{-1} to 5 kV μm^{-1} (a - d). Three independent models of the 10 wt% model set (blue), of the 20 wt% model set (yellow) and of the 30 wt% model set (red) were investigated.

At low electric poling fields of 0.5 kV μm^{-1} (a), only small changes in the order param-

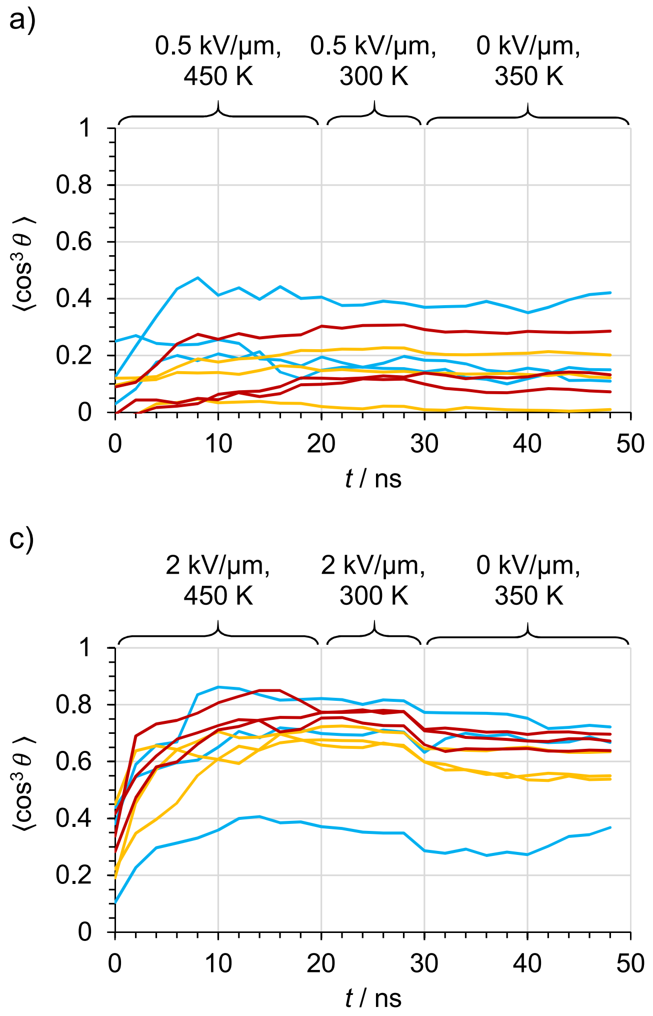

Figure S2: Test simulations of 10 wt% (blue), 20 wt% (yellow) and 30 wt% (red) C3 in PMMA with electric field strengths ranging from 0.5 kV µm^{-1} to 5 kV µm^{-1} (a - d). Subfigure c) shows one low-concentration model (blue), which does not reach an order parameter value comparable to the other eight models.

eter values are visible. All $\langle\cos^3\theta\rangle$ values remain below 0.5. Applying a stronger electric field of $1\,\mathrm{kV\,\mu m^{-1}}$ (b), the order parameter values are increased, but still on a low level, ranging between 10 % and 50 %. After 20 ns of MD simulation, no clear indication of an equilibrium alignment is given. Electric field strengths of $2\,\mathrm{kV\,\mu m^{-1}}$ (c) result in more and better aligned chromophores with order parameter values in the range of 50 % to 70 % after relaxation, except for one low concentration model with a maximum order parameter of 0.4 throughout the simulation. Regardless of the one apparent not-equilibrated model, the chromophores appear to be equilibrated in the electric field and the polymer matrix after 20 ns, because the slope after 10 ns is notable smaller than before. At even stronger electric fields of $5\,\mathrm{kV\,\mu m^{-1}}$ (d), the orientational polarization of the chromophores is accomplished even faster and the resulting order parameter values have a smaller spread. In conclusion a field strength of $5\,\mathrm{kV\,\mu m^{-1}}$ is chosen, to ensure an optimal equilibration with regard to a limited simulation time of 20 ns for the first poling step.

S4 Conventions and Units of the Hyperpolarizability

Various conventions and units are available for β and the T-convention[S2] is used with the following unit conversion

$$\beta^{\mathrm{MKS\,(I)}} = \beta^{\mathrm{esu}} \times \mathrm{statvolt/cm^4} \times 4.1917 \times 10^{-10}\,\mathrm{m^4/V}\text{[S3]} \qquad \text{(S3)}$$

to obtain r_{33} in terms of meters per volt. Additionally, the total hyperpolarizability β_{tot} is utilized, obtained from DFT calculations, where

$$\beta_{\mathrm{tot}} = \frac{5}{3} \times \beta_{||} \qquad \text{(S4)}$$

and $\beta_{||}$ is the hyperpolarizability along the dipole moment.[S2,S4] In some publications β is assumed to be β_{zzz}, i. e. the largest tensor component in most cases ($\beta = \beta_{\mathrm{tot}} \approx \beta_{zzz}$),[S1,S5,S6]

because the experimentally measured macroscopic tensor $\boldsymbol{\beta}$ can be quite complicated and a reasonable and practical simplification can be made when only one component needs to be considered.[S1] In our work, we consistently employ β_{tot}. For comparison, static and wavelength dependent β values from DFT calculations are tabulated later on (c. f. Table S2).

S5 Relation of Microscopic Properties to a Macroscopic Quantity, the Electro-optic Coefficient r_{33}

The EO coefficient r_{33} is related to the macroscopic second-order susceptibility $\chi^{(2)}_{zzz}$ and the microscopic, molecular hyperpolarizability β by[S7,S8]

$$r_{33} = \frac{2\chi^{(2)}_{zzz}}{n_z^4} = \frac{2N_c \beta f_0 f_\lambda^2 \langle \cos^3\theta \rangle}{n_z^4} \tag{S5}$$

where N_c is the number density of chromophores, f_0 and f_λ are local field factors, $\langle \cos^3\theta \rangle$ is the order parameter of the chromophores and n_z is the refractive index with respect to the polar axis.

The local field factors (LFFs) f_0 and f_λ in eq (S5) provide corrections to the incident electric field caused by fields arising from charges in the surrounding environment.[S5] The LFF f_0 is the so called Onsager LFF relating the local electric field to the applied electric field.[S7] For spherical cavities Onsager derived[S9]

$$f_0 = \frac{\varepsilon_0 (n_\lambda^2 + 2)}{n_\lambda^2 + 2\varepsilon_0} \tag{S6}$$

where ε_0 is the permittivity (measured at the conditions of the EO experiment) and n_λ is the refractive index at the wavelength of the EO experiment.[S7] In the main manuscript and the supporting information, all values of the permittivity are related to the vacuum permittivity and are expressed without a unit. It is important not to confuse the low-

frequency / zero frequency permittivity with the vacuum permittivity. Throughout the manuscript and supporting information the low-frequency / zero frequency permittivity is referred to as ε_0 because for traditional reasons. The Lorenz–Lorentz LFF f_λ is described by

$$f_\lambda = \frac{n_\lambda^2 + 2}{3} = \frac{\varepsilon_\lambda + 2}{3} \tag{S7}$$

and relates the local optical field of the host–guest system to the external optical field.[S7] The refractive index at wavelength λ (wavelength of the EO experiment) is given as n_λ. The relation $n_\lambda^2 = \varepsilon_\lambda$ is used and the permittivity at the wavelength of the EO experiment is calculated as follows.[S10] The Lorenz–Lorentz LFF is taking electric field induced electron polarization into account and the Onsager LFF includes dipole reorientation.[S11] These LFFs both rely on strongly simplified models, but are often used to account for the local effects on the microscopic scale providing the transfer of these effects to the macroscopic susceptibility.[S11–S13]

In the main manuscript (e. g. Table 3), there are two values for the electro-optic tensor element presented $\left(r_{33} \text{ and } r_{33}^{\mathrm{PCM}}\right)$, which are estimated based on two different approaches. The first approach is the one already published by Tu et al.[S10] and Zhang et al.[S14] including the above mentioned local field factors. The second approach is a newly devised method, in which the traditional local field factors are implicitly accounted for by (hyper)polarizabilites (e. g. β^{PCM}) calculated in a polarizable continuum model (PCM)

$$r_{33}^{\mathrm{PCM}} = \frac{2N_\mathrm{c}\beta^{\mathrm{PCM}}\langle\cos^3\theta\rangle}{(n_z^{\mathrm{PCM}})^4} \tag{S8}$$

Results of both approaches are compared to experimental investigations and also related to the previously published results. All previously published equations and their modifications are outlined and compared in the next paragraphs.

The permittivity at the wavelength of the EO experiment ε_λ for the host–guest system

$$\varepsilon_\lambda = 1 + 4\pi\left(\chi_{\mathrm{p}}^{(1)} + \chi_{\mathrm{c}}^{(1)}\right) \tag{S9}$$

is approximated by the first-order susceptibilities of the host polymer $\chi_{\mathrm{p}}^{(1)}$ and that for the guest proportion $\chi_{\mathrm{c}}^{(1)}$.[S10,S11] The susceptibility of the chromophore proportion in the host

$$\chi_{\mathrm{c}}^{(1)} = N_{\mathrm{c}}\alpha_{\mathrm{c,iso}} f_\lambda \tag{S10}$$

is calculated using the number density of chromophores N_{c}, the molecular isotropic polarizability of the chromophore $\alpha_{\mathrm{c,iso}}$ and the Lorenz–Lorentz LFF f_λ.[S10] If polarizabilities are calculated with the polarizable continuum model (PCM) method, the Lorenz–Lorentz LFF f_λ becomes obsolete.

The first-order susceptibility for the host $\chi_{\mathrm{p}}^{(1)}$ is estimated using the susceptibility of the pure polymer $\chi_{\mathrm{p},0}^{(1)}$ by[S10]

$$\chi_{\mathrm{p}}^{(1)} = \frac{N_{\mathrm{p}}}{N_{\mathrm{p},0}}\chi_{\mathrm{p},0}^{(1)} = \frac{N_{\mathrm{p}}}{N_{\mathrm{p},0}}\frac{\varepsilon_{\lambda,\mathrm{p},0} - 1}{4\pi} \tag{S11}$$

with the number densities of the repeating units (RU) of the host polymer N_{p} and of the corresponding pure polymer material $N_{\mathrm{p},0}$, respectively. The susceptibility of the pure polymer $\chi_{\mathrm{p},0}^{(1)}$ is expressed in terms of the permittivity at the wavelength of the EO experiment, compare eq (S9). The number density values for the host polymer and the pure polymer (N_{p} and $N_{\mathrm{p},0}$) are calculated using

$$N_{\mathrm{p}} = \frac{\rho_{\mathrm{p}} N_{\mathrm{A}}}{M_{\mathrm{p}}} \quad \text{or} \quad N_{\mathrm{p},0} = \frac{\rho_{\mathrm{p},0} N_{\mathrm{A}}}{M_{\mathrm{p},0}} \tag{S12}$$

where N_{A} is Avogadro's number, M_{p} or $M_{\mathrm{p},0}$ is the molar mass of the host–guest system or pure polymer, ρ_{p} or $\rho_{\mathrm{p},0}$ is the density of the host–guest system or pure polymer,

respectively. Insertion of eq (S10) and (S11) into eq (S9) and rearrangement yields for the LFF approximation (previous method, Lorenz–Lorentz LFF f_λ is replaced by eq (S7))

$$\varepsilon_\lambda = \frac{1 + \frac{N_\mathrm{p}}{N_\mathrm{p,0}}\left(\varepsilon_{\lambda,\mathrm{p},0} - 1\right) + 2x}{1 - x} \quad \text{with} \quad x = \frac{4\pi N_\mathrm{c}\alpha_\mathrm{c,iso}}{3} \tag{S13a}$$

and for the PCM method

$$\varepsilon_\lambda^\mathrm{PCM} = 1 + \frac{N_\mathrm{p}}{N_\mathrm{p,0}}\left(\varepsilon_{\lambda,\mathrm{p},0} - 1\right) + 4\pi N_\mathrm{c}\alpha_\mathrm{c,iso}^\mathrm{PCM} \tag{S13b}$$

The refractive index of pure PMMA at the wavelength of the EO experiment $n_{\lambda,\mathrm{p},0}$ is used to calculate the permittivity of the pure polymer

$$\varepsilon_{\lambda,\mathrm{p},0} = n_{\lambda,\mathrm{p},0}^2 \tag{S14}$$

S6 Refractive Index Estimation of a Host–Guest Model

The refractive index (RI) in the direction of the polar axis (here: z-axis) is calculated by

$$n_z = \sqrt{n_{z,0}^2 + 4\pi N_\mathrm{c}\alpha_\mathrm{c} f_\lambda \left[L_2\left(\frac{\mu f_\mathrm{p} E_\mathrm{p}}{kT}\right) - \frac{1}{3}\right]} \tag{S15}$$

$$\text{with} \quad n_{z,0}^2 = 1 + 4\pi\left(\chi_\mathrm{p}^{(1)} + N_\mathrm{c}\alpha_\mathrm{c} f_\lambda\right) \tag{S16}$$

and taken from Ref S10. The total RI is composed of the RI of the not-poled material $n_{z,0}$ and the additional contribution of the polar aligned chromophore guest molecules (second term under the square root). The latter one consists of the number density of chromophores N_c, their isotropic polarizability α_c, the Lorenz–Lorentz local field factor f_λ and the second-order Langevin function $L_2(x)$. The Langevin function depends on the molecular dipole moment μ, the local field factor of the poling field f_p, the poling field strength E_p, Boltzmann's constant k and the absolute temperature T. The RI of the

non-poled material $n_{z,0}$ is composed of the susceptibility of the host polymer $\chi_{\mathrm{p}}^{(1)}$ and the susceptibility of the incorporated guest molecules (compare eq (S9)). Additionally,

$$\Delta n_z^2 = 4\pi N_{\mathrm{c}}(\alpha_{||} - \alpha_{\perp}) \left(\langle \cos^2 \theta \rangle - \frac{1}{3} \right) \tag{S17}$$

is taken from Ref S15 and the second term under the square root in eq (S15) is replaced with the definition of Δn_z^2 in eq (S17). Using this substitution the theoretical value of the Langevin function ($L_2(x)$, rigid, ideal gas model) can be replaced with the $\langle \cos^2 \theta \rangle$ value calculated from MD simulations (implicitly taking care of guest-guest / guest-host interactions). Furthermore, eq (S17) contains more specific polarizabilities representations, calculated as

$$\alpha_{||} := \alpha_{zz} \tag{S18}$$

and

$$\alpha_{\perp} := \alpha_{xx} = \alpha_{yy} \tag{S19}$$

Here, the polarizability orthogonal to the dipole moment (main axis of the molecular system) $\alpha_{\perp}$ is calculated as

$$\alpha_{\perp} := \frac{\alpha_{xx} + \alpha_{yy}}{2} \tag{S20}$$

because the chromophore C3 is not symmetric regarding rotation (the equivalence $\alpha_{xx} = \alpha_{yy}$ is not satisfied). Insertion of eq (S17) in eq (S15) leads to

$$n_z = \sqrt{n_{z,0}^2 + 4\pi N_{\mathrm{c}} \left(\alpha_{\mathrm{c},||} - \alpha_{\mathrm{c},\perp} \right) f_{\lambda} \left[\langle \cos^2 \theta \rangle - \frac{1}{3} \right]} \tag{S21}$$

$$\text{with} \quad n_{z,0}^2 = 1 + 4\pi \left(\chi_{\mathrm{p}}^{(1)} + N_{\mathrm{c}} \alpha_{\mathrm{c,iso}} f_{\lambda} \right) = \varepsilon_{\lambda} \tag{S22}$$

where $\alpha_{\text{c,iso}}$ represents the molecular isotropic polarizability of the chromophore to describe the polarizability of the not-poled host–guest material. If PCM calculations are performed, local field factors become obsolete because local field effects are implicitly included in the polarizabilities, the eqs (S21) and (S22) are reduced to

$$n_z^{\text{PCM}} = \sqrt{\left(n_{z,0}^{\text{PCM}}\right)^2 + 4\pi N_{\text{c}} \left(\alpha_{\text{c},||}^{\text{PCM}} - \alpha_{\text{c},\perp}^{\text{PCM}}\right) \left[\langle \cos^2\theta \rangle - \frac{1}{3}\right]} \quad \text{(S23)}$$

$$\text{with} \quad \left(n_{z,0}^{\text{PCM}}\right)^2 = 1 + 4\pi \left(\chi_{\text{p}}^{(1)} + N_{\text{c}}\alpha_{\text{c,iso}}^{\text{PCM}}\right) = \varepsilon_\lambda^{\text{PCM}} \quad \text{(S24)}$$

S7 Optical Properties

Table S1 lists properties of PMMA needed for the calculation of the permittivity of the host–guest material at the wavelength of the EO experiment (ε_λ). In the experimental setup a wavelength of 970 nm was chosen due to the availability of cost-effective possible lasing media like Ti:Saphire, InGaAs or Nd:YAG for that wavelength. Table S2 shows additional components of the (hyper)polarizability tensors for the C3 chromophore.

Table S1: PMMA related properties from literature.

Property	Value	Ref	Notes
n_λ	1.4769	S16,S17	$\lambda = 970$ nm
ε_0	3.6	S18	measured at a frequency of 50 Hz, assumed to be static
f_0	1.60	SI, eq (S6)	

Table S2: Elements of the polarizability tensor α and the hyperpolarizability tensor β in T-convention[a] for the C3 chromophore (indices: $0 =$ static value, $\lambda = 970\,\text{nm}$).

Polarizability α (in 10^{-24} esu) and Hyperpolarizability β (in 10^{-30} esu) of C3 … from the main manuscript		
	in vacuo	PCM
$\alpha_{xx,\lambda}$	38.4	43.4
$\alpha_{yy,\lambda}$	58.8	66.3
$\alpha_{zz,\lambda}$	162.3	240.2
$\beta_{\text{tot},\lambda}$	490.1	1068.6
… additional values for comparison		
$\alpha_{xx,0}$	37.8	45.9
$\alpha_{yy,0}$	57.2	69.2
$\alpha_{zz,0}$	132.0	195.0
$\beta_{\text{tot},0}$	256.0	574.9
$\beta_{\|\|,0}$	153.6	345.0
$\beta_{\|\|,\lambda}$	294.0	641.2
$\beta_{zzz,0}$	−238.5	−535.3
$\beta_{zzz,\lambda}$	−455.4	−989.8

[a] Ref S2: Origin and meaning of different well established conventions.

S8 Relation to Previous Studies

Tu et al.[S10] and Zhang et al.[S14] were the first authors who devised an approach to relate MD results (supported by quantum mechanical calculations) with a macroscopic response quantity, i. e. the electro-optic coefficient, the r_{33} value. However, both authors did not convert β from CGS (in cm^4/statvolt) to SI units (in m^4/V).[S3] Moreover, both authors used $\beta_{||}$ in B-convention as the quantity to include the molecular nonlinear response. In this paper, $\beta_{\text{tot}} = \frac{5}{3}\beta_{||} = \beta_{\text{vec}}$ is used, because β_{vec} is the most commonly measured quantity in experiments.[S2,S4] In the present case the equality $\beta_{\text{tot}} = \beta_{\text{vec}}$ is given, because the charge transfer is unidirectional and parallel to the molecular dipole moment, which is aligned along the z-direction.[S4] The calculated hyperpolarizability value is not further modified in the present study, so that the T-convention is used.

In the present paper, one approach was to calculate the Onsager local field factor based on the well-known properties of the pure polymer host with the traditional equation (see eq (S6)) to enable a straightforward comparison to existing (experimental) studies (e. g. of Tu et al.[S10] and Zhang et al.[S14]) and for future easily accessible comparisons. The fitting procedure to approximate the Onsager local field factor devised by Tu et al.[S10] and Zhang et al.[S14] can be applied to small chromophore molecules in very dilute (liquid state) systems (appropriate to the rigid ideal gas model). However, it seems not to be adequate to describe the herein presented host–guest systems with larger, more sterically demanding chromophore molecules with stronger intermolecular interactions, in a polymer matrix.

The r_{33} values presented by Tu et al.[S10] and Zhang et al.[S14] $r_{33}^{as\,pub}$ were modified

$$r_{33}^{mod} = r_{33}^{as\,pub} \times 4.1917 \times 10^{-2}\,\text{cm}^4/\text{V} \times 2 \times \frac{5}{3} \times \frac{f_0}{f_0^{as\,pub}} \tag{S25}$$

according to the aforementioned unit conversion (first factor), convention $\left(2 \times \frac{5}{3}\right)$ and local field factor approximation $\left(f_0/f_0^{as\,pub}\right)$ to be comparable to the values presented in this paper. The Onsager local field factor f_0 (see eq (S6)) is calculated based on the refractive

index at the wavelength of the EO experiment (in the case of Tu et al.[S10]: $n_\lambda = 1.4893$ at $\lambda = 1900\,\text{nm}$) and the zero frequency permittivity ($\varepsilon_0 = 3.6$, see Table S1). The local field factor is 1.60 for PMMA in the present study ($\lambda = 970\,\text{nm}$) and for Tu et al.[S10] at a wavelength of $\lambda = 1900\,\text{nm}$ the local field factor has a value of 1.61. For amorphous polycarbonate (APC) the value is 1.57. The RI, $n_\lambda = 1.586$, was taken from Ref S14 and $\varepsilon_0 = 2.9$ was taken from a substance data sheet for thin-film polymeric host–guest systems, Ref S19.

S9 Sample Preparation, Poling Procedure, and Teng–Man Measurement

Sample Preparation A mixture of chromophore C3 at 15 wt% in host polymer PMMA (PLEXIGLAS® 8N, Röhm), was dissolved in 1,2-dichloroethane (Sigma-Aldrich, ACS reagent grade ≥99.0 %) at 5 wt% solids in solution. Indium tin oxide (ITO) coated soda-lime glass (25 mm × 25 mm, 0.7 mm thickness) was used as substrate. To reduce the occurrence of short circuits, a 50 nm Al_2O_3 layer was deposited onto the substrates via atomic layer deposition with trimethylaluminum and water as precursors at 90 °C and $<10^{-3}$ mbar in a Savannah 200 system (Cambridge Nanotech). An ITO strip of roughly 3 mm was left uncoated for electrical contact. The substrates were washed with acetone, then isopropanol in an ultrasonic bath and treated in an ozone generator to remove residual organic impurities. Polymer solutions were filtered through a 0.1 µm PTFE-filter (Yeti, infochroma) and deposited onto the substrate via spin-coating at 500 rpm (50 rpm/s acceleration, 40 s) to produce a polymer layer thickness of around 1 µm. Prepared samples were dried in a vacuum oven at 90 °C for 1 h and then at 60 °C overnight to remove residual solvent. The layer thickness of the polymer was determined by a Dektak 8 stylus profilometer (Veeco).

Poling Procedure Samples were poled with a corona poling setup with the ITO layer acting as the bottom electrode and a needle mounted above the sample as the top electrode. Samples were heated to 135 °C and a voltage of 4 kV was applied with a distance of 25 mm between needle and polymer surface. After 30 min of poling, the heating plate was turned off and the polymer was allowed to cool to room temperature before voltage was switched off.

Teng–Man Measurement A metal electrode was deposited onto the poled polymer layers via vapor deposition (aluminum, $\leq$100 nm) and wires attached to both electrodes. The sample is mounted into the Teng–Man setup and a polarized 970 nm laser beam (laser diode controller LDC 205C, temperature controller TED 200C, Thorlabs) directed at the ITO side of the sample with an incidence angle of 45°. The beam is reflected by the metal electrode behind the polymer layer, phase retarded through a Soleil-Babinet compensator and filtered through an analyzer, before arriving at the photodetector. The photodetector is connected to a lock-in amplifier and preamplifier (Model SR810 DSP lock-in amplifier + Model SR552 bipolar preamplifier, Stanford Research Systems). An AC voltage with a frequency of 1 kHz and an amplitude of 10 V is applied to the electrodes of the sample with a signal generator (pulse / function generator 8021, Kontron Elektronik), which is also connected to the lock-in amplifier for reference. The setup was calibrated using z-cut $LiNbO_3$ (Bontek) and z-cut $LiTaO_3$ (Korth).

The Teng–Man method is known to produce large errors due to internal reflections on the various optical interfaces inside the stack and absorption effects on the transparent conductive oxide layer and metal electrode. To take these into account, the methodology and code presented in Ref S20 and in Ref S21 by Park et al. was used. Additionally, an aperture was placed in front of the photodetector to minimize scattered light. Based on the layer thicknesses, refractive indices and extinction coefficients (from refractiveindex.info[S17]) of our sample (Table S3), a value and error of $r_{33} = (20.9 \pm 5.1)\,\mathrm{pm\,V^{-1}}$ was obtained.

Table S3: Layer system and refractive indices (RIs, TCO: transparent conductive oxide).

Layer (top to bottom)	Thickness $d/\mu m$	(Complex) RI at $\lambda = 970\,nm$	Ref for RI
Soda lime glass	1000	1.517	S22
TCO, In_2O_3–SnO_2	0.1	1.275 + 0.062i	S23
Buffer layer, Al_2O_3	0.05	1.617	S24
15 wt% C3 in PMMA	(1.069 ± 0.050)	1.595	Table 4 (manuscript) and Section S10 (SI)
Al	0.1	1.218 + 8.163i	S25

S10 Ellipsometry Measurements

Host–guest materials were spin-coated on glass or Si wafer as substrate. Three samples were measured and the average values are presented in the main manuscript. Ellipsometry measurements were performed using a Sentech SE850 ellipsometer in a spectral range of 192 nm to 1700 nm with angles of incidence between 50° and 70°. Data evaluation was conducted with the "CompleteEASE" from J. A. Woollam. Figure S3 shows the different applied optical models to estimate the refractive index.

Model 1: The optical properties of glass were measured using a clean glass plate. It was described with a Cauchy model with four free parameters. The PMMA samples were described using a Cauchy model with five set parameters and one free parameter. Model 2: The PMMA samples with chromophores were described using a b-spline with 0.15 eV resolution. The non-uniform thickness was taken into account in the model as a free parameter. Model 3: The thickness of SiO_2 was determined using a clean Si reference sample. Later on the thickness of SiO_2 was fixed. The optical properties of the layers were described using a b-spline function with 0.1 eV resolution.

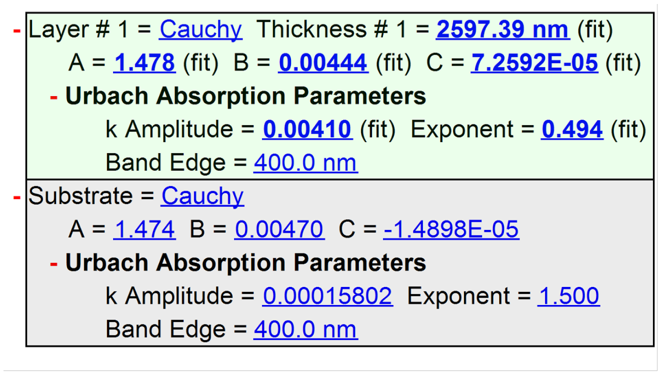

a) Model 1: Glass substrate + pure PMMA.

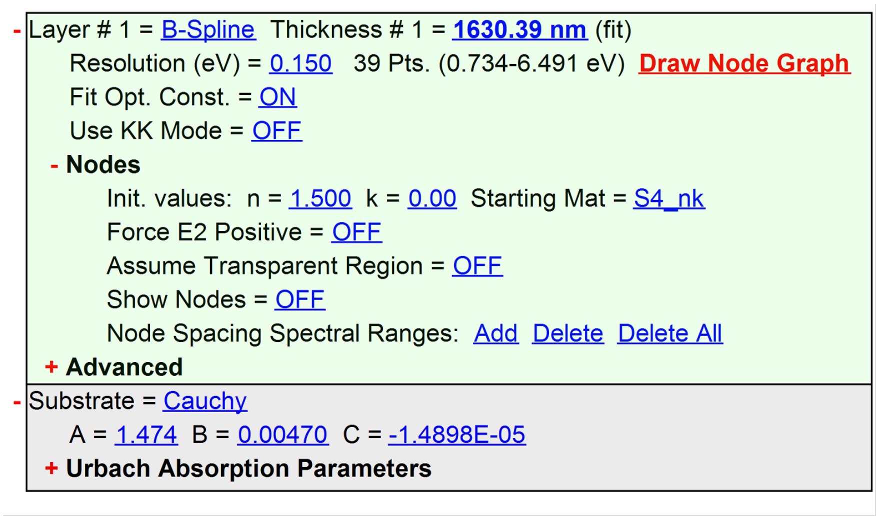

b) Model 2: Glass substrate + PMMA with chromophores.

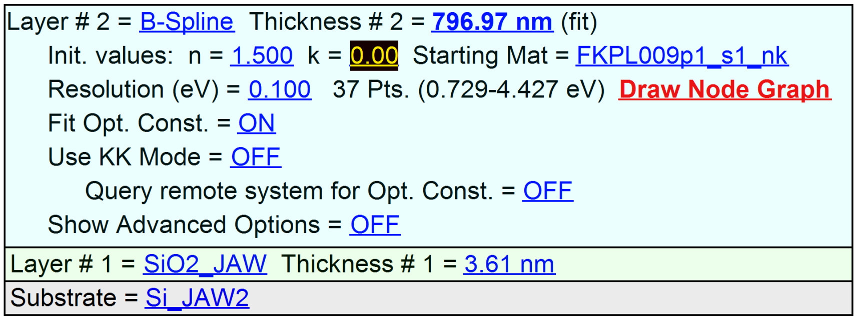

c) Model 3: Si substrate + oxide layer + PMMA with chromophores.

Figure S3: Parameters and setups of the different optical models used in the evaluation of the refractive index.

References

(S1) Burland, D. M.; Miller, R. D.; Walsh, C. A. Second-order nonlinearity in poled-polymer systems. *Chem. Rev.* **1994**, *94*, 31–75.

(S2) Willetts, A.; Rice, J. E.; Burland, D. M.; Shelton, D. P. Problems in the comparison of theoretical and experimental hyperpolarizabilities. *J. Chem. Phys.* **1992**, *97*, 7590–7599.

(S3) Shi, R. F.; Garito, A. F. In *Characterization techniques and tabulations for organic nonlinear optical materials*; Kuzyk, M. G., Dirk, C. W., Eds.; Optical engineering; Dekker: New York, 1998; pp 1–36.

(S4) Kanis, D. R.; Ratner, M. A.; Marks, T. J. Design and construction of molecular assemblies with large second-order optical nonlinearities. Quantum chemical aspects. *Chem. Rev.* **1994**, *94*, 195–242.

(S5) Williams, D. In *Electronic and photonic applications of polymers*; Bowden, M. J., Turner, S. R., Eds.; Advances in chemistry; American Chemical Society: Washington, DC, 1988; Vol. 218; pp 297–330.

(S6) Dalton, L. R.; Sullivan, P. A.; Bale, D. H. Electric field poled organic electro-optic materials: state of the art and future prospects. *Chem. Rev.* **2010**, *110*, 25–55.

(S7) Robello, D. R.; Dao, P. T.; Phelan, J.; Revelli, J.; Schildkraut, J. S.; Scozzafava, M.; Ulman, A.; Willand, C. S. Linear polymers for nonlinear optics. 2. Synthesis and electrooptical properties of polymers bearing pendant chromophores with methylsulfonyl electron-acceptor groups. *Chem. Mater.* **1992**, *4*, 425–435.

(S8) Dalton, L. R.; Steier, W. H.; Robinson, B. H.; Zhang, C.; Ren, A.; Garner, S.; Chen, A.; Londergan, T.; Irwin, L.; Carlson, B.; Fifield, L.; Phelan, G.; Kincaid, C.; Amend, J.; Jen, A. From molecules to opto-chips: organic electro-optic materials. *J. Mater. Chem.* **1999**, *9*, 1905–1920.

(S9) Onsager, L. Electric Moments of Molecules in Liquids. *J. Am. Chem. Soc.* **1936**, *58*, 1486–1493.

(S10) Tu, Y.; Zhang, Q.; Ågren, H. Electric field poled polymeric nonlinear optical systems: molecular dynamics simulations of poly(methyl methacrylate) doped with disperse red chromophores. *J. Phys. Chem. B* **2007**, *111*, 3591–3598.

(S11) Prasad, P. N.; Williams, D. J. *Introduction to nonlinear optical effects in molecules and polymers*; A Wiley-Interscience publication; John Wiley & Sons: New York and Chichester and Brisbane and Toronto and Singapore, 1991.

(S12) Kuzyk, M. G.; Poga, C. In *Molecular nonlinear optics*; Zyss, J., Kelley, P., Liao, P. F., Eds.; Quantum electronics - Principles and applications; Academic Press: Boston, 1994; pp 299–337.

(S13) Mukamel, S. In *Molecular nonlinear optics*; Zyss, J., Kelley, P., Liao, P. F., Eds.; Quantum electronics - Principles and applications; Academic Press: Boston, 1994; pp 1–46.

(S14) Zhang, Q.; Tu, Y.; Tian, H.; Ågren, H. Molecular dynamics simulations of polycarbonate doped with Lemke chromophores. *J. Phys. Chem. B* **2007**, *111*, 10645–10650.

(S15) Wu, J. W. Birefringent and electro-optic effects in poled polymer films: steady-state and transient properties. *J. Opt. Soc. Am. B* **1991**, *8*, 142.

(S16) Zhang, X.; Qiu, J.; Li, X.; Zhao, J.; Liu, L. Complex refractive indices measurements of polymers in visible and near-infrared bands. *Appl. Opt.* **2020**, *59*, 2337–2344.

(S17) Polyanskiy, M. N. Refractiveindex.info database of optical constants. *Sci. Data* **2024**, *11*, 94.

(S18) Brandrup, J.; Immergut, E. H.; Grulke, E. A.; Abe, A.; Bloch, D. R. *Polymer Handbook*, 4th ed.; A Wiley-Interscience publication; Wiley: New York, 1999.

(S19) Covestro Deutschland AG *Product range Apec®*; 2019.

(S20) Park, D. H.; Lee, C. H.; Herman, W. N. Analysis of multiple reflection effects in reflective measurements of electro-optic coefficients of poled polymers in multilayer structures. *Opt. Express* **2006**, *14*, 8866–8884.

(S21) Park, D. H. Characterization of linear electro-optic effect of poled organic thin films. Dissertation, University of Maryland, College Park, United States – Maryland, 2008.

(S22) Kamptner, A.; Scharber, M. C.; Schiek, M. Accurate determination of the uniaxial complex refractive index and the optical band gap of polymer thin films to correlate their absorption strength and onset of absorption. *ChemPhysChem* **2024**, *25*, e202400233.

(S23) Minenkov, A.; Hollweger, S.; Duchoslav, J.; Erdene-Ochir, O.; Weise, M.; Ermilova, E.; Hertwig, A.; Schiek, M. Monitoring the electrochemical failure of indium tin oxide electrodes via operando ellipsometry complemented by electron microscopy and spectroscopy. *ACS Appl. Mater. Interfaces* **2024**, *16*, 9517–9531.

(S24) Zhukovsky, S. V.; Andryieuski, A.; Takayama, O.; Shkondin, E.; Malureanu, R.; Jensen, F.; Lavrinenko, A. V. Experimental demonstration of effective medium approximation breakdown in deeply subwavelength all-dielectric multilayers. *Phys. Rev. Lett.* **2015**, *115*, 177402.

(S25) McPeak, K. M.; Jayanti, S. V.; Kress, S. J. P.; Meyer, S.; Iotti, S.; Rossinelli, A.; Norris, D. J. Plasmonic films can easily be better: rules and recipes. *ACS Photonics* **2015**, *2*, 326–333.
